# Supplementary material for: Projections of incident atherosclerotic cardiovascular disease and incident type 2 diabetes across evolving statin treatment guidelines and recommendations: A modelling study
Source: PLoS Med. 2020 Aug 26;17(8):e1003280. doi: 10.1371/journal.pmed.1003280 (PMC7449387; doi:10.1371/journal.pmed.1003280)
Supplement: S1 Table — ASCVD, atherosclerotic cardiovascular disease. (DOCX) [file pmed.1003280.s001.docx]

| **Population** | **S0(t) at 10 Years** | | | **Mean score** | | **Individual Score** | |
| --- | --- | --- | --- | --- | --- | --- | --- |
| *Participants not taking hypertensive medications* | | | | | | | |
| Black women | | 0.9533 | 86.61 | | = 17.114 × ln(age) + 0.94 × ln(TC) - 18.92 × ln(HDL-C) + 4.475 × ln(age) × ln(HDL-C) + | |  |
| White women | | 0.9665 | -29.18 | | 27.82 × ln(SBP) - 6.087 × ln(age) × ln(SBP) (+ 0.691 if current smoker) (+ 0.874 if diabetes)  = - 29.799 × ln(age) + 4.884 × ln(age)2 + 13.54 × ln(TC) - 3.114 × ln(age) × ln(TC) -13.578 × | |  |
|  | |  |  | | ln(HDL-C) + 3.149 × ln(age) × ln(HDL-C) + 1.957 × ln(SBP) (+ 7.574 - 1.665 × ln(age) if | |  |
|  | |  |  | | current smoker) (+ 0.661 if diabetes) | |  |
| Black men | | 0.8954 | 19.54 | | = 2.469 × ln(age) + 0.302 × ln(TC) - 0.307 × ln(HDL-C) + 1.809 × ln(SBP) (+ 0.549 if current | |  |
|  | |  |  | | smoker) (+ 0.645 if diabetes) | |  |
| White men | | 0.9144 | 61.18 | | = 12.344 × ln(age) + 11.853 × ln(TC) - 2.664 × ln(age) × ln(TC) - 7.99 × ln(HDL-C) + 1.769 × | |  |
|  | |  |  | | ln(age) × ln(HDL-C) + 1.764 × ln(SBP) (+ 7.837 - 1.795 × ln(age) if current smoker) (+ 0.658 | |  |
|  | |  |  | | if diabetes) | |  |
| *Participants taking hypertensive medications* | | | | | | |  |
| Black women | | 0.9533 | 86.61 | | = 17.114 × ln(age) + 0.94 × ln(TC) - 18.92 × ln(HDL-C) + 4.475 × ln(age) × ln(HDL-C) + | |  |
| White women | | 0.9665 | -29.18 | | 29.291 × ln(SBP) - 6.432 × ln(age) × ln(SBP) (+ 0.691 if current smoker) (+ 0.874 if diabetes)  = - 29.799 × ln(age) + 4.884 × ln(age)2 + 13.54 × ln(TC) - 3.114 × ln(age) × ln(TC) -13.578 × | |  |
|  | |  |  | | ln(HDL-C) + 3.149 × ln(age) × ln(HDL-C) + 2.019 × ln(SBP) (+ 7.574 - 1.665 × ln(age) if | |  |
|  | |  |  | | current smoker) (+ 0.661 if diabetes) | |  |
| Black men | | 0.8954 | 19.54 | | = 2.469 × ln(age) + 0.302 × ln(TC) - 0.307 × ln(HDL-C) + 1.916 × ln(SBP) (+ 0.549 if current | |  |
|  | |  |  | | smoker) (+ 0.645 if diabetes) | |  |
| White men | | 0.9144 | 61.18 | | = 12.344 × ln(age) + 11.853 × ln(TC) - 2.664 × ln(age) × ln(TC) - 7.99 × ln(HDL-C) + 1.769 × | |  |
|  | |  |  | | ln(age) × ln(HDL-C) + 1.797 × ln(SBP) (+ 7.837 - 1.795 × ln(age) if current smoker) (+ 0.658 | |  |
|  | |  |  | | if diabetes) | |  |

TC = total cholesterol

HDL-C = high-density lipoprotein cholesterol

SBP = Systolic blood pressure

Adapted from ACC/AHA Recommendation on the Assessment of Cardiovascular Risk working group et al., 2013
