# Supplementary material for: Projections of incident atherosclerotic cardiovascular disease and incident type 2 diabetes across evolving statin treatment guidelines and recommendations: A modelling study
Source: PLoS Med. 2020 Aug 26;17(8):e1003280. doi: 10.1371/journal.pmed.1003280 (PMC7449387; doi:10.1371/journal.pmed.1003280)
Supplement: S2 Table — (DOCX) [file pmed.1003280.s002.docx]

| **Population Parameters** | **Males (95% CI)** | **Females (95% CI)** |
| --- | --- | --- |
| **ASCVD** |  |  |
| Overall ASCVD annual incidence rate/10,000 person-years* | | |
| 40-44** | 50 (30-70) | 20 (10-30) |
| 45-50 | 50 (30-70) | 20 (10-30) |
| 51-55 | 70 (50-90) | 30 (20-40) |
| 56-60 | 60 (50-70) | 40 (10-70) |
| 61-65 | 110 (90-130) | 10 (50-80) |
| 66-70 | 150 (130-170) | 100 (70-130) |
| 71-75 | 200 (160-240) | 130 (110-150) |
| Statin-ASCVD relative risk | 0.64 (0.55, 0.75) | 0.84 (0.64 − 1.10) |
| **T2D** |  |  |
| Overall T2D annual incidence rate/ 10,000 person-years* | | |
| 40-44** | 160 (120-220) | 140 (100-180) |
| 45-50 | 160 (120-220) | 140 (100-180) |
| 51-55 | 170 (140-190) | 160 (100-180) |
| 56-60 | 160 (130-190) | 140 (100-180) |
| 61-65 | 130 (100-150) | 150 (120-180) |
| 66-70 | 140 (110-170) | 130 (110-170) |
| 71-75 | 120 (90-150) | 110 (80-140) |
| Statin-T2D relative risk | 1.11, 1.32, 1.55 | 1.11, 1.32, 1.55 |
| **Non-ASCVD mortality** |  |  |
| Overall Non-ASCVD mortality rate/10,000 person-years*** | | |
| 40-44 | 20 (16-24) | 10 (8-12) |
| 45-50 | 30 (24-36) | 20 (16-24) |
| 51-55 | 50 (40-60) | 30 (24-36) |
| 56-60 | 60 (50-70) | 40 (32-48) |
| 61-65 | 80 (70-90) | 50 (40-60) |
| 66-70 | 100 (90-110) | 70 (60-80) |
| 71-75 | 160 (140-180) | 120 (100-140) |
| Statin-Non-ASCVD mortality relative risk | 1.0 | 1.0 |
