# Supplementary material for: Projections of incident atherosclerotic cardiovascular disease and incident type 2 diabetes across evolving statin treatment guidelines and recommendations: A modelling study
Source: PLoS Med. 2020 Aug 26;17(8):e1003280. doi: 10.1371/journal.pmed.1003280 (PMC7449387; doi:10.1371/journal.pmed.1003280)
Supplement: S3 Table — (DOCX) [file pmed.1003280.s003.docx]

| **Age (5-year) sex-specific parameters** | **Sources of data** | **Metric estimated** |
| --- | --- | --- |
| Statin Eligibility | NHANES | 10-year ASCVD risk |
| ASCVD parameters |  |  |
| ASCVD incidence overall | REGARDS | Annual incidence rate |
| Statin-ASCVD RR | Cholesterol Treatment Trialists’ Collaboration[11] | RR |
| T2D parameters |  |  |
| T2D incidence overall | REGARDS | Annual incidence rate |
| Statin-T2D RR | Past meta-analysis [12] | RR |
| Non-ASCVD mortality parameters |  |  |
| Total non-ASCVD mortality overall | National Center for Health Statistics | Annual mortality rate |
| Statin-non-ASCVD mortality RR | NA | RR |
| Statin parameters |  |  |
| Prevalence of statin discontinuation | REGARDS | Prevalence proportion |
