# Supplementary material for: Projections of incident atherosclerotic cardiovascular disease and incident type 2 diabetes across evolving statin treatment guidelines and recommendations: A modelling study
Source: PLoS Med. 2020 Aug 26;17(8):e1003280. doi: 10.1371/journal.pmed.1003280 (PMC7449387; doi:10.1371/journal.pmed.1003280)
Supplement: S4 Table — ASCVD, atherosclerotic cardiovascular disease. (DOCX) [file pmed.1003280.s004.docx]

|  | | |
| --- | --- | --- |
| **Ages** | **Males**  **% (N)** | **Females**  **% (N)** |
| **ASCVD risk: 5-7.5%** | N = 3,523,107 | N = 4,566,692 |
| 40-44 | 7.9 (278,325) | 2.6 (118,734) |
| 45-50 | 14.3 (503,804) | 8.3 (379,035) |
| 51-55 | 26.9 (947,716) | 8.3 (379,035) |
| 56-60 | 38.6 (1,359,919) | 16.6 (758,071) |
| 61-65 | 11.1 (391,065) | 39.9 (1,822,110) |
| 66-70 | 1.2 (42,277) | 23.0 (1,050,339) |
| 71-75 | 0.0 (0.0) | 1.3 (59,367) |
| **ASCVD risk: 7.5-10%** | N = 4,036,059 | N = 2,764,377 |
| 40-44 | 2.5 (100,901) | 0.6 (16,586) |
| 45-50 | 10.7 (431,858) | 2.4 (66,345) |
| 51-55 | 20.1 (811,248) | 6.2 (171,391) |
| 56-60 | 26.4 (1,065,520) | 9.6 (265,380) |
| 61-65 | 29.4 (1,186,601) | 30.7 (848,664) |
| 66-70 | 10.7 (431,858) | 40.5 (1,119,573) |
| 71-75 | 0.2 (8,072) | 10.0 (276,439) |
| **ASCVD risk: ≥10%** | N = 18,182,735 | N = 8,556,581 |
| 40-44 | 0.8 (145,462) | 0.6 (51,339) |
| 45-50 | 3.4 (618,213) | 1.5 (128,349) |
| 51-55 | 7.1 (1,290,974) | 2.2 (188,245) |
| 56-60 | 13.1 (2,381,938) | 5.1 (436,386) |
| 61-65 | 25.4 (4,618,415) | 9.9 (847,102) |
| 66-70 | 27.1 (4,927,521) | 29.0 (2,481,408) |
| 71-75 | 23.1 (4,200,212) | 51.7 (4,423,752) |
