# Supplementary figures and images for: Projections of incident atherosclerotic cardiovascular disease and incident type 2 diabetes across evolving statin treatment guidelines and recommendations: A modelling study
Source: PLoS Med. 2020 Aug 26;17(8):e1003280. doi: 10.1371/journal.pmed.1003280 (PMC7449387; doi:10.1371/journal.pmed.1003280)

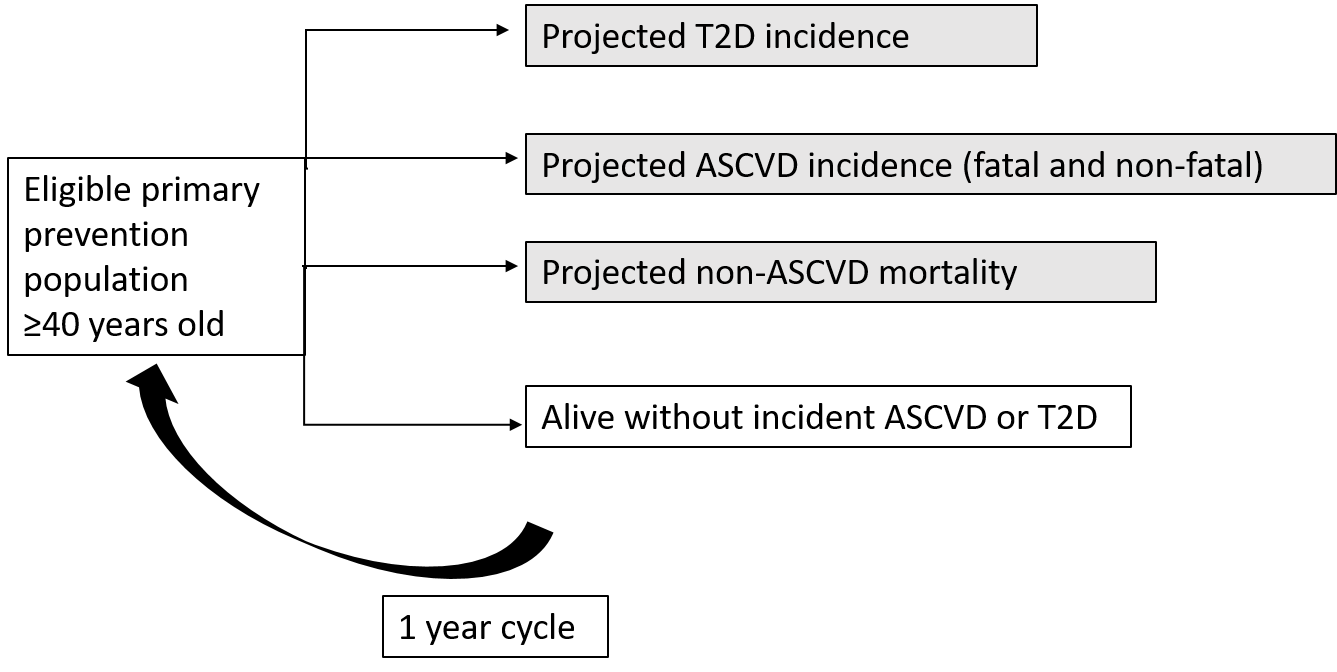

Supplement: S1 Fig — Rectangles correspond to disease states, and arrows represent the allowed transitions. Absorbing states are shaded. ASCVD, atherosclerotic cardiovascular disease; T2D, type 2 diabetes. (TIF) [file pmed.1003280.s005.tif]

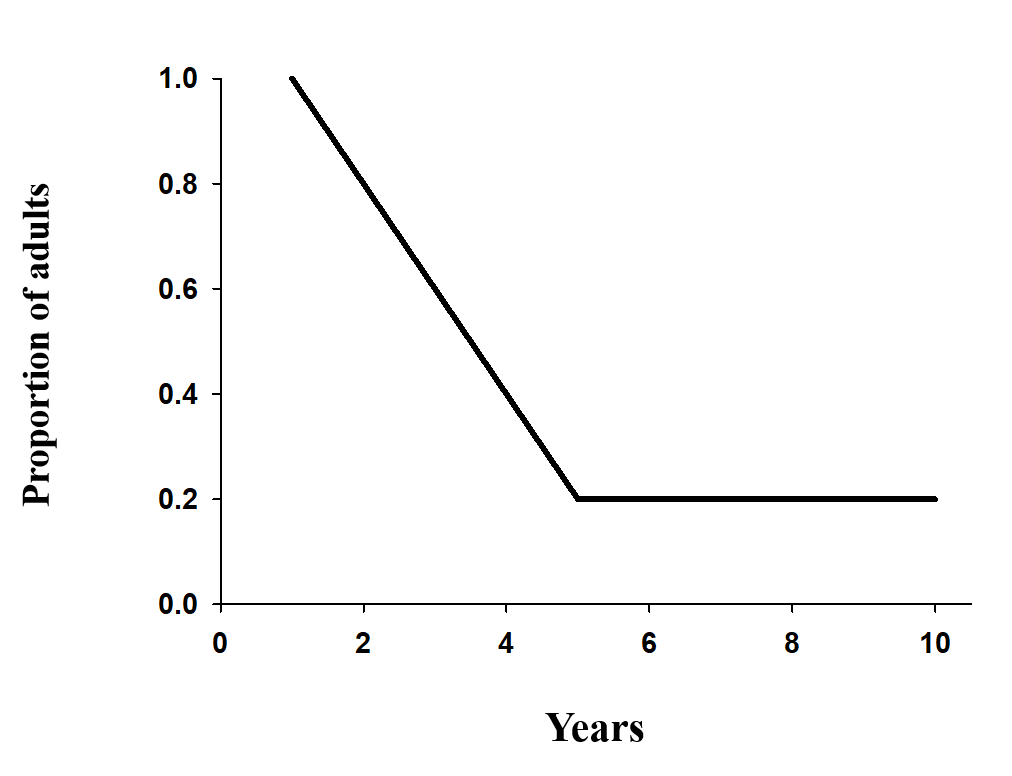

Supplement: S2 Fig — (TIF) [file pmed.1003280.s006.tif]

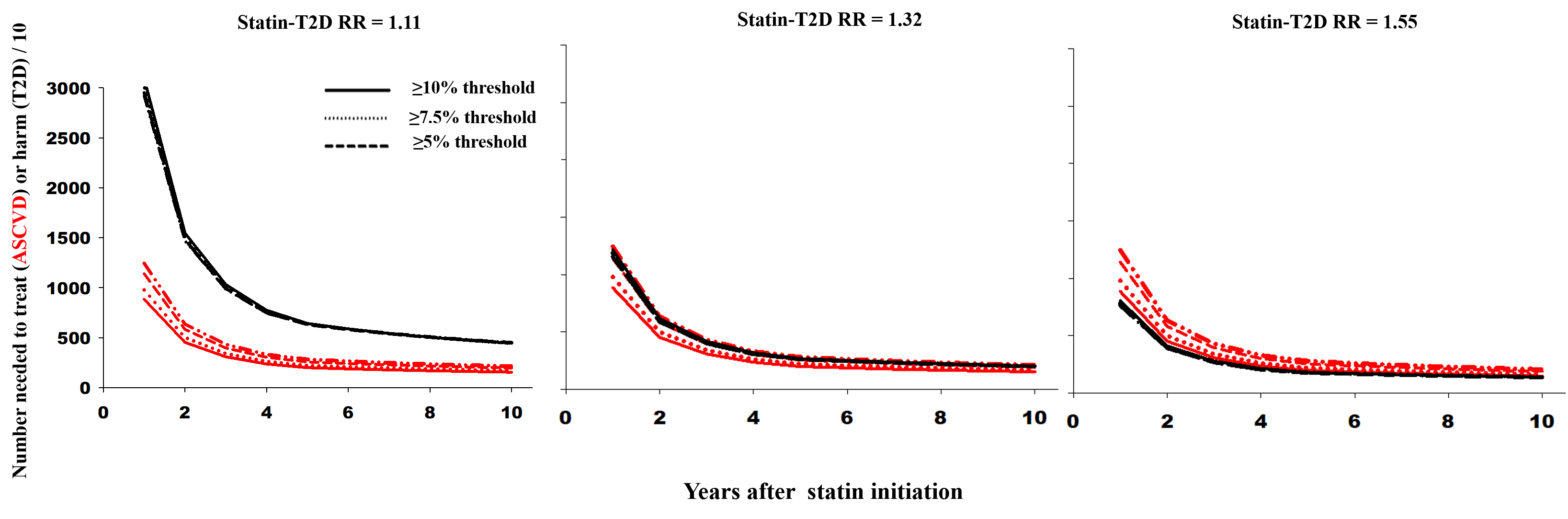

Supplement: S3 Fig — NNH, number needed to harm; NNT, number needed to treat. (TIF) [file pmed.1003280.s007.tif]

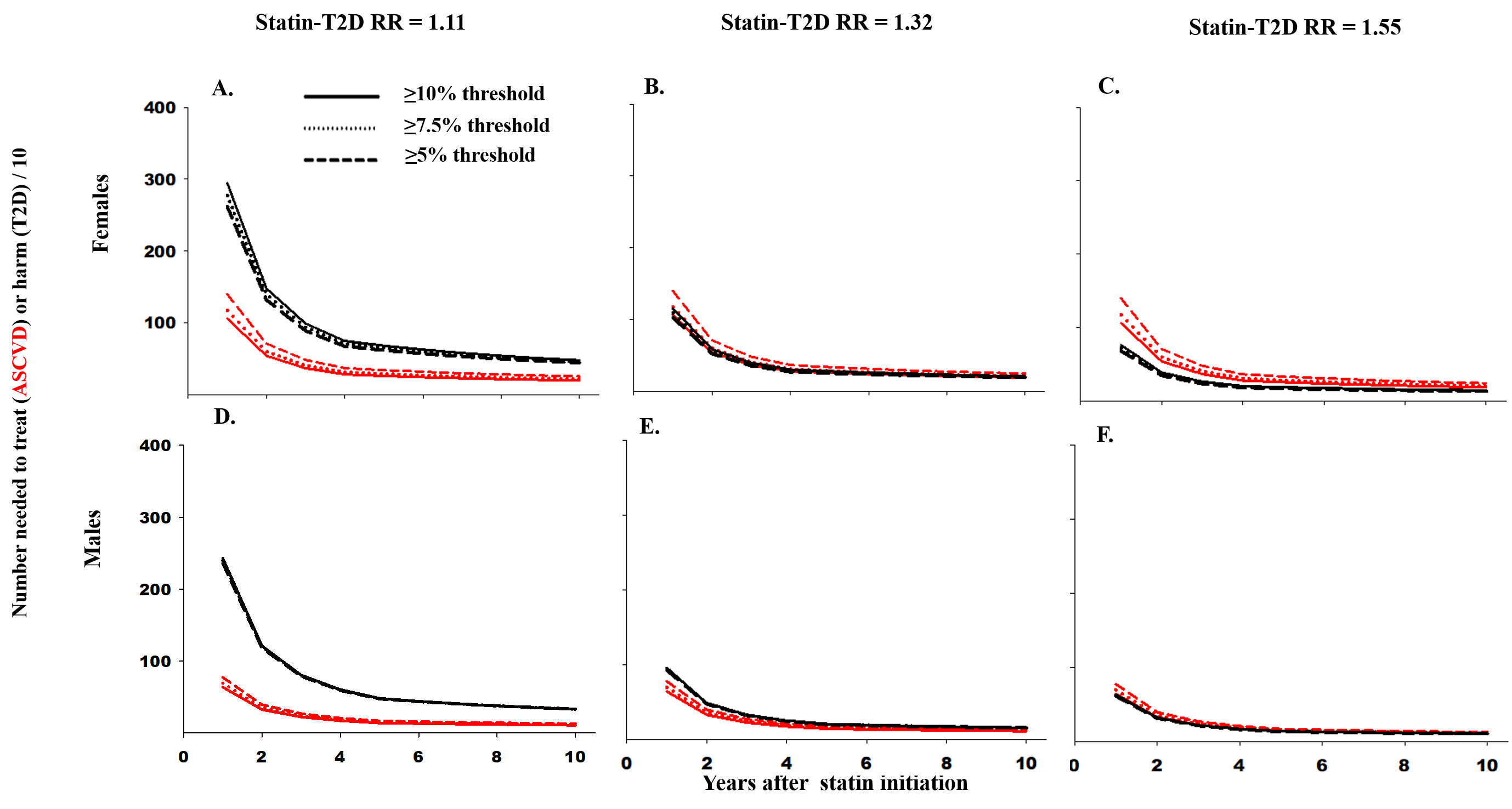

Supplement: S4 Fig — NNT or NNH among females (panels A–C), and males (panels D–F) associated with 3 statin treatment guidelines or recommendations among a projected population of 61,125,042 eligible U.S. African American and white adults in 2014. NNH, number needed to harm; NNT, number needed to treat. (TIF) [file pmed.1003280.s008.tif]

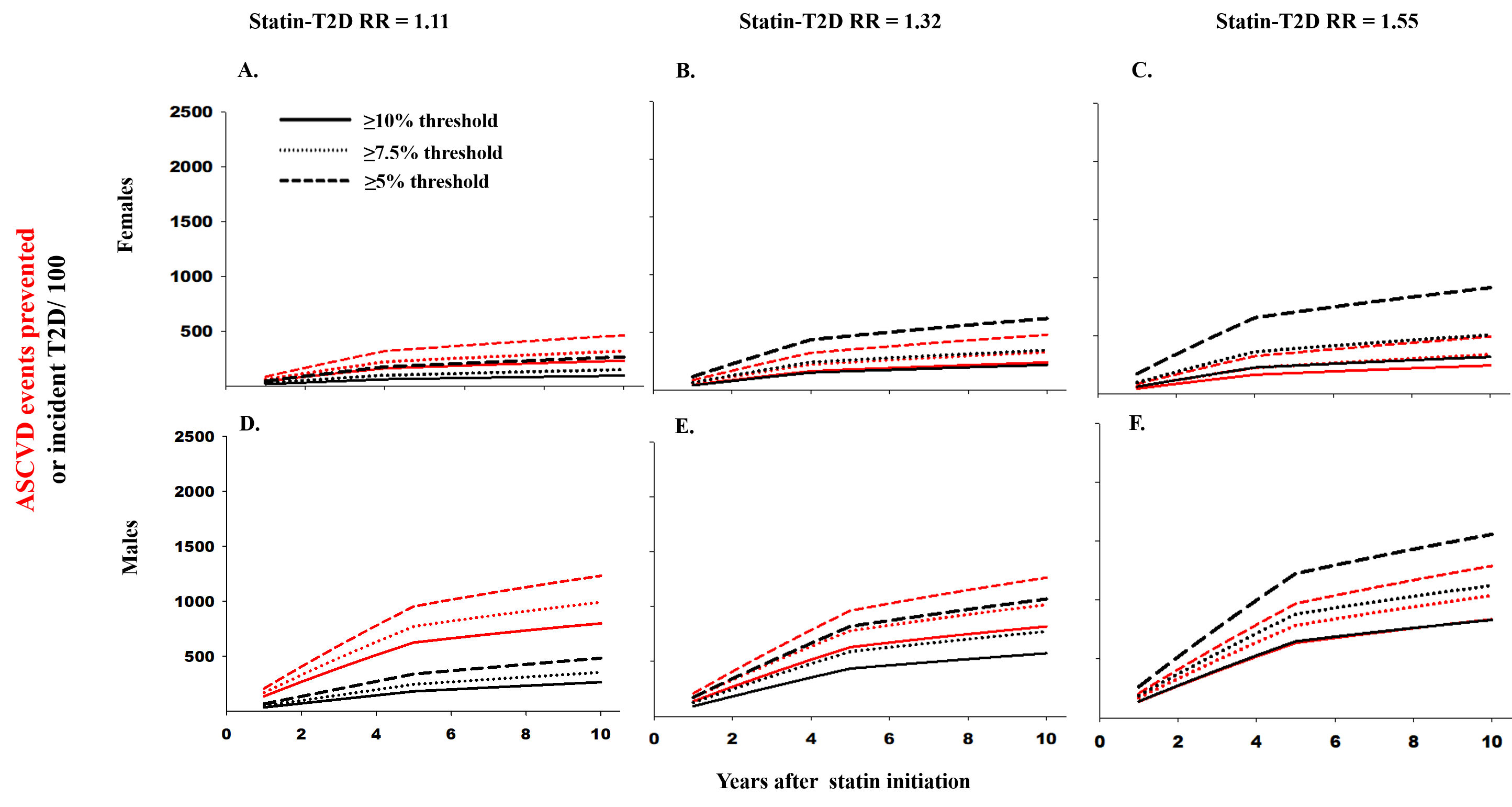

Supplement: S5 Fig — Cumulative number of events of ASCVD and T2D among females (panels A–C) and males (panels D–F) associated with 3 statin treatment guidelines or recommendations from a projected population of 61,125,042 eligible U.S. African American and white adults in 2014. ASCVD, atherosclerotic cardiovascular disease; T2D, type 2 diabetes. (TIF) [file pmed.1003280.s009.tif]

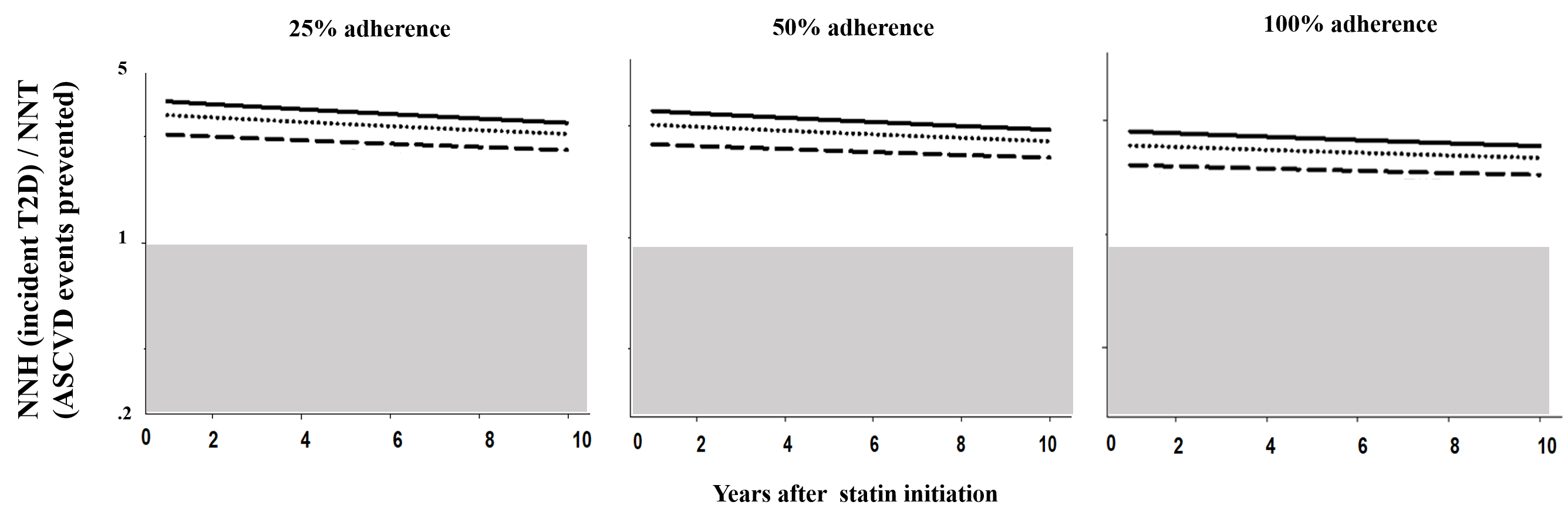

Supplement: S6 Fig — Grey line describes threshold when NNH > NNT. Statin-T2D RR = 1.11. LLH, likelihood to be helped or harmed; NNH, number needed to harm; NNT, number needed to treat; RR, relative risk; T2D, type 2 diabetes. (TIF) [file pmed.1003280.s010.tif]

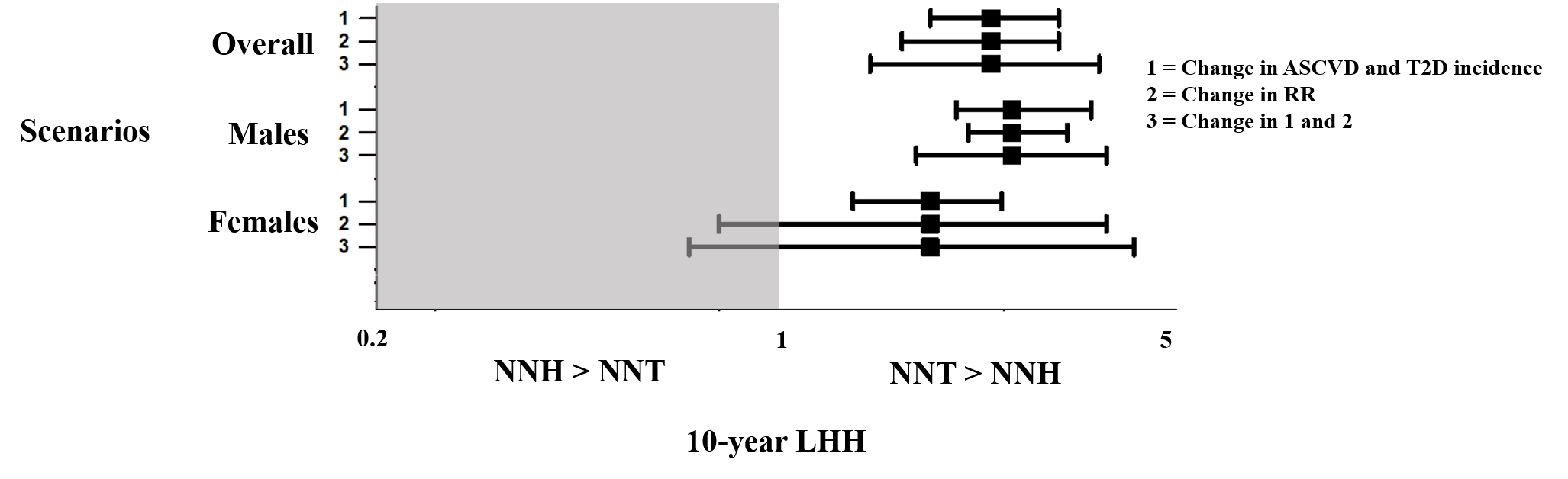

Supplement: S7 Fig — Uncertainty was quantified through 3 PSAs: 2 PSAs that considered uncertainty from each of the input parameters separately and a third PSA that considered uncertainty from each input parameter simultaneously. Gray shading indicates the portions of the uncertainty intervals for which the NNH exceeds the NNT. ASCVD, atherosclerotic cardiovascular disease; LLH, likelihood to be helped or harmed; PSA, probabilistic sensitivity analysis; NNH, number needed to harm; NNT, number needed to treat. (TIF) [file pmed.1003280.s011.tif]

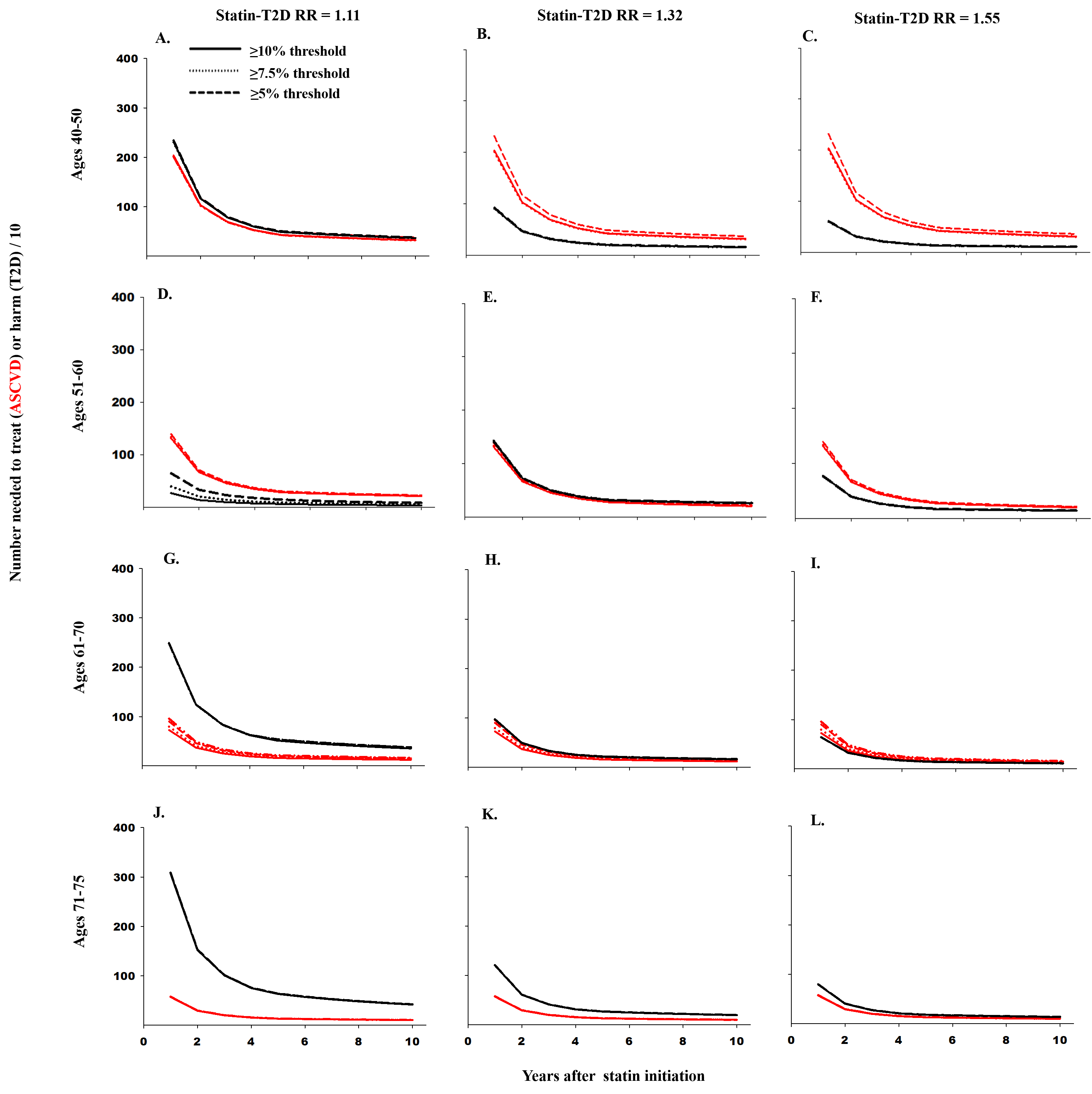

Supplement: S8 Fig — NNT or NNH among 40- to 50-year-olds (panels A–C), 51- to 60-year-olds (panels D–F), 61- to 70-year-olds (panels G–I), and 71- to 75-year-olds (panels J–L) associated with 3 statin treatment guidelines or recommendations among a projected population of 61,125,042 eligible U.S. African American and white adults in 2014. NNH, number needed to harm; NNT, number needed to treat. (TIF) [file pmed.1003280.s012.tif]
