# Supplementary material for: Projections of incident atherosclerotic cardiovascular disease and incident type 2 diabetes across evolving statin treatment guidelines and recommendations: A modelling study
Source: PLoS Med. 2020 Aug 26;17(8):e1003280. doi: 10.1371/journal.pmed.1003280 (PMC7449387; doi:10.1371/journal.pmed.1003280)
Supplement: S1 STROBE checklist — STROBE, Strengthening the Reporting of Observational Studies in Epidemiology. (DOCX) [file pmed.1003280.s013.docx]

|  | Item No | Recommendation | Paragraph and Section |
| --- | --- | --- | --- |
| **Title and abstract** | 1 | (*a*) Indicate the study’s design with a commonly used term in the title or the abstract | Abstract |
|  |  | (*b*) Provide in the abstract an informative and balanced summary of what was done and what was found |  |
| Introduction | | | |
| Background/rationale | 2 | Explain the scientific background and rationale for the investigation being reported | Accumulating evidence has suggested that statins increase the relative risk (RR) of T2D by 5-55%(10-15), with potentially elevated statin-associated T2D risk in females(16), younger populations compared to older populations, and populations with lower LDL-C compared to populations with higher LDL-C.(15) Such findings merit further investigation in light of evolving statin recommendations that target growing proportions of populations for ASCVD primary prevention, for whom the net effects of statins remain incompletely quantified.(17) (Introduction section, 2^nd^ paragraph) |
| Objectives | 3 | State specific objectives, including any prespecified hypotheses | Therefore, this study projected the number of expected ASCVD events prevented and incident cases of T2D incurred in primary prevention populations across three 10-year ASCVD risk-based statin treatment guidelines or recommendations (Introduction section, 2^nd^ paragraph). |
| Methods | | | |
| Study design | 4 | Present key elements of study design early in the paper | A simulation model was conceptualized and then developed in September 2018 to evaluate statin guidelines and recommendations because few available studies (1) were contemporary, (2) spanned ages specified by current guidelines or recommendations, and (3) precisely and validly measured ASCVD and T2D incidence within generalizable male and female multi-ethnic populations with long-term follow-up [21]. This study did not consider costs associated with statins, T2D, or ASCVD (Methods section, 1^st^ paragraph). |
| Setting | 5 | Describe the setting, locations, and relevant dates, including periods of recruitment, exposure, follow-up, and data collection | After conceptualizing the problem, the next step was to identify input data. In an attempt to maximize generalizability, we prioritized studies that included multi-ethnic (non-Hispanic African American and non-Hispanic White; capturing 73% of the U.S. population [22]) male and female statin-eligible adults aged 40-75 years, reflecting the ages specified by the AHA/ACC 2013 and 2018 guidelines [3, 4] (Methods section, 2^nd^ paragraph). |
| Participants | 6 | (*a*) Give the eligibility criteria, and the sources and methods of selection of participants. Describe methods of follow-up | We defined the primary prevention population as 40-75 year old males and females of self-reported non-Hispanic African American or non-Hispanic White race/ethnicity who did not report a doctor or health professional diagnosis of ASCVD, T2D, or type 1 diabetes, who reported never taking cholesterol medications (assessed by self-report and medication inventory), and who had measured fasting LDL-C levels ≤ 190 mg/dl. Race/ethnic-, sex-, -and anti-hypertensive therapy- specific predicted 10-year ASCVD risk for each participant of the primary prevention population was then calculated using the Pooled Cohort Equation ( see **S1 Methods**, **S1 Table**) [24], noting that we restricted our evaluation to statin guidelines or recommendations that used the Pooled Cohort Equation (Methods section, 3^rd^ paragraph). |
|  |  | (*b*) For matched studies, give matching criteria and number of exposed and unexposed |  |
| Variables | 7 | Clearly define all outcomes, exposures, predictors, potential confounders, and effect modifiers. Give diagnostic criteria, if applicable | Incident CHD was defined based on medical records, signs and symptoms, diagnostic cardiac enzymes, or electrocardiographic changes consistent with myocardial infarction or a CHD death (**S2 Table**). Incident stroke was centrally adjudicated by physicians using the World Health Organization definition or by review of final reports from all available neuroimaging studies that were consistent with acute ischemia [27, 28]. Among participants without T2D at baseline who returned to visit 2, T2D incidence was defined by fasting glucose ≥126 mg/dl, non-fasting glucose >200 mg/dl or use of glucose-lowering medication (**S2 Table**). Because the REGARDS study only included participants aged ≥45 years and given the comparability of estimated ASCVD and T2D incidence rates between ages 45-60, we assigned ASCVD and T2D incidence rates estimated in the 45-50 year age group to the 40-44 year age group.  Non-ASCVD mortality rates were obtained from the National Center for Health Statistics [29], which compiled death certificates filed in all 50 states and the District of Columbia. Annual non-ASCVD deaths were defined excluding heart disease (International Statistical classification of Disease, 10^th^ Edition codes [ICD-10]: I00-I09, I11, I13, I20-I51) and cerebrovascular (ICD-10: I60-I69) deaths (Methods section, 4^th^ and 5^th^ paragraph). |
| Data sources/ measurement | 8* | For each variable of interest, give sources of data and details of methods of assessment (measurement). Describe comparability of assessment methods if there is more than one group | Demographic characteristics, 10-year ASCVD risk, and the number of adults eligible for statin therapy initiation were estimated using the biennial, cross-sectional, and nationally representative National Health and Nutrition Examination Survey (NHANES, waves 2007-2014), pooling four waves to ensure sufficient precision [23] (see **S1 Text**). NHANES collects demographic, nutritional, and health status information on a nationally representative probability sample of the U.S. civilian population instituted by the National Center for Health Statistics.  In the absence of nationally representative data measuring ASCVD and T2D incidence using validated protocols capturing undiagnosed disease and mortality [25], we leveraged data from the ongoing Reasons for Geographic and Racial Differences in Stroke (REGARDS) study (data used: 2003-2015). The REGARDS study is a contemporary, population-based, longitudinal cohort study designed to evaluate factors underlying the excess stroke mortality in the southeast versus other regions of the U.S. and among African American versus White adults (see **S1 Text**) [26]  (Methods section, 3^rd^ and 4^th^ paragraphs). |
| Bias | 9 | Describe any efforts to address potential sources of bias | To evaluate possible sources of heterogeneity, we projected the expected benefits and harms of statin treatment by sex and age [18, 40]. To examine the robustness of projections to different adherence patterns, we also projected the expected number of ASCVD events prevented and expected number of excess incident case of T2D incurred under three annual relative decrease in statin use of 25% and 50%, as well as full adherence (i.e. 0% decrease) across 10 years [36]. Finally, we examined the robustness of projections to variation in two sets of study input parameters (sex-specific statin-ASCVD RRs as well as sex- and age-specific ASCVD and T2D incidence rates) by performing three probabilistic sensitivity analyses (PSA) (Methods section, 12^th^ paragraph). |
| Study size | 10 | Explain how the study size was arrived at | Demographic characteristics, 10-year ASCVD risk, and the number of adults eligible for initiation of statin therapy were estimated using the biennial, cross-sectional, and nationally representative National Health and Nutrition Examination Survey (NHANES, waves 2007-2014), pooling four waves to ensure sufficient precision (Methods section, 3^rd^ paragraph). |
| Quantitative variables | 11 | Explain how quantitative variables were handled in the analyses. If applicable, describe which groupings were chosen and why | We contrasted statin-associated ASCVD and T2D incidence across three statin treatment guidelines or recommendations using state-transition Markov simulation models.(33) The state-transition Markov simulation model projected annual estimates of T2D and ASCVD incidence based on the above-defined parameters. For each annual cycle, statin-eligible populations could either remain alive and non-diseased or transition to having T2D, an ASCVD event, or a non-ASCVD death. As we were comparing incidence of T2D and ASCVD, once cohort members had T2D, an ASCVD event or non-ASCVD death, cohort members were removed from the population and were no longer simulated (see example in **S1 Figure**).(33) The one-year cycle was repeated 10 times for each age- (one-year) and sex-specific group and for each intervention scenario to project statin-associated 10-year ASCVD and T2D incidence. (Methods section, 9^th^ paragraph). |
| Statistical methods | 12 | (*a*) Describe all statistical methods, including those used to control for confounding | Using these scenarios, we projected the absolute benefit of statin treatment as the number needed to treat (NNT) to prevent one ASCVD event (1/ [statin-associated ASCVD risk difference]), with smaller values indicating a larger absolute risk reduction; hereafter NNT will be referred to as number needed to benefit (NNB). Absolute harm of statin treatment was calculated as the number needed to harm (NNH[1/statin-association T2D risk difference]) to cause one excess incident case of T2D, with larger values indicating that more people needed to receive statin treatment to cause one excess incident case of T2D.(35) In addition, we estimated the likelihood to be helped or harmed (LHH), defined as the ratio of the NNH to NNB; LLH values < 1 indicated that the number of incident cases of T2D incurred exceeded the number of ASCVD events prevented.(36) Statistical analyses were performed using STATA (College Station, TX), TreeAge Healthcare Pro Suite 2018 (TreeAge Software, Williamstown, MA), and SAS (Cary, NC). (Methods section, 11^th^ paragraph). |
|  |  | (*b*) Describe any methods used to examine subgroups and interactions |  |
|  |  | (*c*) Explain how missing data were addressed |  |
|  |  | (*d*) If applicable, explain how loss to follow-up was addressed |  |
|  |  | (*e*) Describe any sensitivity analyses |  |
| Results | | |  |
| Participants | 13* | (a) Report numbers of individuals at each stage of study—eg numbers potentially eligible, examined for eligibility, confirmed eligible, included in the study, completing follow-up, and analysed | Supplement |
|  |  | (b) Give reasons for non-participation at each stage |  |
|  |  | (c) Consider use of a flow diagram |  |
| Descriptive data | 14* | (a) Give characteristics of study participants (eg demographic, clinical, social) and information on exposures and potential confounders | When weighted to the 2014 non-institutionalized, civilian U.S. population, our primary prevention population consisted of 61,125,042 adults (**Table 1).** |
|  |  | (b) Indicate number of participants with missing data for each variable of interest |  |
|  |  | (c) Summarise follow-up time (eg, average and total amount) |  |
| Outcome data | 15* | Report numbers of outcome events or summary measures over time | As the proportion of adults eligible for statin therapy increased, so did the number of ASCVD events prevented over 10 years (**Figure 1**). The ≥10% ASCVD risk threshold guideline was projected to prevent the fewest ASCVD events (N=103,009) (**Figure 1, panel A**), whereas the ≥5.0% ASCVD risk threshold was projected to prevent the largest number of ASCVD events (N=169,370). (pg. 11) |

| Main results | 16 | (*a*) Give unadjusted estimates and, if applicable, confounder-adjusted estimates and their precision (eg, 95% confidence interval). Make clear which confounders were adjusted for and why they were included | When assuming a statin associated T2D risk of 1.11, 10-year NNH projections were consistent across guidelines or recommendations, ranging from 444-446 (**Fig** **S3**). These projections suggested that for all statin treatment guidelines or recommendations, the number of ASCVD events prevented was at least twice as large as the number of incident cases of T2D incurred (LHH range 2.26 – 2.90; NNB range 155-215) (**Fig 1, panels A and D; Fig S3**). However, projections of absolute and relative harm were sensitive to the assumed statin associated T2D RR. When the statin associated T2D RR was increased to 1.32, NNHs decreased to 198-202 and the relative benefits of statin treatment decreased (LHH range: 1.03-1.30; NNB range 155-209) (**Fig 1, panels B and E; Fig S3**). Sensitivity analyses that varied adherence to statin treatment resulted in proportional decreases in the number of ASCVD events prevented and incident cases of T2D incurred (**Fig S6**), although results for LHH projections remained consistent (Results section, 3^rd^ paragraph). |
| --- | --- | --- | --- |
|  |  | (*b*) Report category boundaries when continuous variables were categorized |  |
|  |  | (*c*) If relevant, consider translating estimates of relative risk into absolute risk for a meaningful time period |  |
| Other analyses | 17 | Report other analyses done—eg analyses of subgroups and interactions, and sensitivity analyses | We next examined the benefits and harms of statin treatment by sex. Across all scenarios, females received lower absolute benefits and incurred a higher relative burden of adverse events when compared to males (**Fig 2, Fig S4**). The absolute and relative benefits of statin treatment guidelines and recommendations also were more variable in females compared to males. For example, when assuming a statin-T2D RR=1.11, one ASCVD event was prevented for every 196-254 females treated (LHH range: 1.74-2.40; NNH range 430-478) across the three statin treatment guidelines or recommendations. For males, one ASCVD event was prevented for every 110-131 males treated (LHH range: 2.55-3.00; NNH range 331-334) across the three statin treatment guidelines or recommendations. When assuming a statin-T2D RR-1.32, LHH ranged from 0.77-1.1 in females, but remained above 1 (LHH range: 1.18-1.43) for males. Consistent with our findings, PSA indicated that estimates in females were more uncertain than estimates in males or the total population (**Fig S7**) (Results section, 4^th^ paragraph). |
| Discussion | | | |
| Key results | 18 | Summarise key results with reference to study objectives | We projected that 13 to 28 million non-Hispanic African American and White adults would be newly eligible for statin treatment, among whom one ASCVD event would be prevented for every 155-197 adults treated. Benefits of statin treatment were even more pronounced in males and older populations compared to females and younger populations. (Discussion section, 1^st^ paragraph). |
| Limitations | 19 | Discuss limitations of the study, taking into account sources of potential bias or imprecision. Discuss both direction and magnitude of any potential bias | Despite many strengths, there are limitations that merit consideration….(Discussion section, 6^th^ paragraph). |
| Interpretation | 20 | Give a cautious overall interpretation of results considering objectives, limitations, multiplicity of analyses, results from similar studies, and other relevant evidence | In conclusion, this simulation study adds to a growing body of literature examining the net effects of statins in primary prevention populations. Our results suggest that the highest relative burden of T2D occurred among female and younger adult populations and highlight areas where additional clinical and public health research is needed. (Discussion section, last paragraph). |
| Generalisability | 21 | Discuss the generalisability (external validity) of the study results | Although it remains difficult to anticipate the magnitude by which differences in population characteristics affect estimates of statin-associated T2D risk, prior studies reporting potential heterogeneity by age, sex, and health characteristics support evaluating a range of potential RR estimates rather than relying on a single estimate.(10, 15, 51) (Discussion section, 5^th^ paragraph). |
| Other information | | | |
| Funding | 22 | Give the source of funding and the role of the funders for the present study and, if applicable, for the original study on which the present article is based |  |

*Give information separately for exposed and unexposed groups.

**Note:** An Explanation and Elaboration article discusses each checklist item and gives methodological background and published examples of transparent reporting. The STROBE checklist is best used in conjunction with this article (freely available on the Web sites of PLoS Medicine at http://www.plosmedicine.org/, Annals of Internal Medicine at http://www.annals.org/, and Epidemiology at http://www.epidem.com/). Information on the STROBE Initiative is available at http://www.strobe-statement.org.
