# Supplementary material for: Projections of incident atherosclerotic cardiovascular disease and incident type 2 diabetes across evolving statin treatment guidelines and recommendations: A modelling study
Source: PLoS Med. 2020 Aug 26;17(8):e1003280. doi: 10.1371/journal.pmed.1003280 (PMC7449387; doi:10.1371/journal.pmed.1003280)
Supplement: S1 Text — (DOCX) [file pmed.1003280.s014.docx]

**S1 Text**

We leveraged multi-ethnic (non-Hispanic African American and non-Hispanic White) and sex-specific data from the National Health and Nutrition Examination Survey (NHANES) and the Reasons for Geographic and Racial Differences in Stroke (REGARDS) study as well as statin associated relative risks (RRs) from past meta-analyses to assess changes in type 2 diabetes (T2D) and atherosclerotic cardiovascular disease (ASCVD) incidence from changes in 10-year ASCVD risk thresholds.

### A. Data sources and inputs

The project was conducted by assembling and integrating contemporary and validated data from NHANES, REGARDS, and past meta-analyses into a Markov model.

#### A.1.NHANES

Data from NHANES were used to construct the initial statin-eligible population (population size and baseline characteristics). NHANES is a series of cross-sectional surveys and physical examinations conducted biennially to assess the health and nutritional status of the U.S. population and is publically available at <https://wwwn.cdc.gov/nchs/nhanes/Default.aspx> (1). NHANES collects demographic, nutritional, and health status information on a nationally representative probability sample of the U.S. civilian population instituted by the National Center for Health Statistics (NCHS). Participants are randomly selected through a complex, multistage cluster sampling probability design. Young children, older adults, African Americans, and Mexican Americans are oversampled at each survey to provide sufficient numbers to support analysis in these underrepresented populations. All analyses used sample weights to produce estimates generalizable to the original sampling frame (2) For this study, we used data from three recent NHANES population cross-sections, conducted in 2007-2014.

Exclusion criteria

NHANES participants self-reporting coronary heart disease (CHD), angina, stroke (all types), participants classified as having diabetes (T2D and type 1 diabetes [T1D]), participants with triglyceride levels >400 mg/dl, participants with LDL-C levels ≥190 mg/dl, and participants with ASCVD risk scores unable to be calculated (i.e. missing data) were excluded from the study. Additionally, only African American and White populations between the ages of 40-75 years were considered in the analysis to overlap with ages eligible for statin treatment under the ACC/AHA treatment recommendations.

#### A.2.REGARDS

Data from REGARDS were used to estimate ASCVD and T2D incidence and statin discontinuation. REGARDS is a national, population-based, longitudinal study designed to evaluate factors underlying the excess stroke burden in the southeastern US and among African Americans (3).To gain access to REGARDS data, a manuscript proposal was submitted to [REGARDSAdmin@uab.edu](mailto:REGARDSAdmin@uab.edu). Potentially eligible REGARDS participants were identified from commercially available nationwide lists of U.S. residents with a recruitment goal of including 30% of participants from the “Stroke Belt” (North Carolina, South Carolina, Georgia, Tennessee, Mississippi, Alabama, Louisiana, and Arkansas). Eligible participants were sent an initial mailing, followed by a telephone call and a subsequent in-home visit. Between January 2003 and October 2007, REGARDS enrolled 30,239 non-institutionalized African American and White adults aged ≥45 years. The second in-home visit took place from May 2013 to Dec 2016.

Exclusion criteria

REGARDS participants self-reporting MI/CHD, angina, stroke (all types), transient ischemic attack, participants classified as having T2D or type 1 diabetes, participants with triglycerides >400 mg/dl, or participants with LDL-C ≥190 mg/dl were excluded from the analyses. Only participants between the ages 45-75 years were considered in the analysis to overlap with ages eligible for statin treatment under the ACC/AHA treatment recommendations (minimum age for REGARDS is 45 years). Because the REGARDS study only included participants from ages 45 years and older and our observation for comparable ASCVD and T2D incidence rates between ages 45-60, we assigned incidence rates estimated in the 45-50 year age group to the 40-44 year age group.

####

#### A.3. Data inputs

##### A.3.1.Data from NHANES

Data from NHANES were used to obtain baseline characteristics of the study population; 10-year risk of ASCVD; and history of T2D, ASCVD, and statin medication use. Questionnaires were used to assess prevalence of ASCVD, demographic information, smoking status, and blood pressure treatment. The mean of final two of three blood pressure measurements from the medical examination were used to assess systolic blood pressure. Fasting blood samples were used for lipid analyses to measure total cholesterol (TC), high-density lipoprotein cholesterol (HDL-C), and triglycerides. Low-density lipoprotein cholesterol (LDL-C) levels were calculated using the Friedewald equation.(4) The prevalence of T2D was assessed by participant-report of a doctor or health professional diagnosis of T2D (yes/no) or report of T2D medication use. The prevalence of current use of statins was defined by self-report of ever taking cholesterol medications.

##### A.3.2.Data from REGARDS

Incident and prevalent ASCVD and T2D were estimated to inform ASCVD and T2D parameters and were ascertained from the REGARDS study by participant report or adjudicated by physicians.

MI/CHD

Self-reported MI prevalence, which was used as an exclusion criterion when estimating ASCVD incidence, was ascertained using the following questions: (1) “Has a doctor or any other health professional ever told you that you had a myocardial infarction or heart attack?” (2) “Have you ever had a coronary artery bypass surgery, such as a graft, CABG, or a bypass procedure on the arteries of your heart?” and (3) “Have you ever had an angioplasty or stenting of a coronary artery with or without placing a coil in the artery to keep it open?” All questions were scored “yes”, “no”, “don’t know”, and “refused” (3). MI/CHD was defined as a yes response to any one of those three questions.

Incident CHD events were ascertained during follow-up. Participants were contacted by telephone every six months to assess hospitalizations, emergency department visits, overnight stays in nursing homes or rehabilitation centers, or death. If suspected heart event was reported, medical records were pursued. MI were adjudicated based on the presence of signs or symptoms suggestive of ischemic; diagnostic cardiac enzymes (rising or falling pattern in cardiac troponin or creatinine phosphokinase-MB isoenzyme concentrations over ≥ six hours with a peak concentration greater than twice the upper limit of normal); and ECG changes consistent with ischemia or MI (5). In the case where a participant died outside the hospital, interviews with family members or other proxies, proximal hospitalizations, baseline medical history, death certificates, and the National Death Index were used to identify CHD as the underlying cause of death.

Stroke

Prevalent stroke, which was used as an exclusion criterion when estimating ASCVD incidence, was defined as a positive response to either “Were you ever told by a physician that you had a stroke?” or “Were you ever told by a physician you had a mini-stroke or TIA, also known as a transient ischemic attack?” (3).

Similar to the ascertainment of incident MI/CHD, incident stroke was determined during follow-up. Participants were contacted by telephone every six months to assess vital status, identify hospitalizations, emergency department visits, or overnight stays in nursing homes or rehabilitation centers. Reasons for medical encounters were asked and medical records were sought for stroke, TIA, death, unknown reason for hospitalization, or if reason was brain aneurysm, brain hemorrhage, sudden weakness, numbness, trouble speaking, sudden loss of vision, headache, other stroke symptoms (3). Reports of possible incident stroke events were reviewed by a stroke nurse and then reviewed by at least two physician members of a panel of stroke experts in accordance with the World Health Organization definition (6). For proxy reported deaths, interview was conducted with next of kin.

T2D

T2D was ascertained during the first in-home visit (to ascertain prevalent T2D) and second in-home visit (to ascertain incident T2D) using REGARDS investigator defined outcomes (T2D if fasting glucose ≥126 mg/dl, non-fasting glucose >200 mg/dl or taking oral glucose-lowering medication or insulin) (3).

### B. Statin eligibility using Pooled Cohort Risk Equations

To identify statin-eligible populations from NHANES, 10-year ASCVD risk scores were estimated for each member of the primary prevention population using their measured exposures and the equation described below to determine statin treatment assignment.

Using NHANES data, the predicted 10-year risk for ASCVD for each member of the primary prevention population was calculated using the Pooled Cohort risk equations, developed by the ACC/AHA Task Force on Practice Recommendations (7). Separate equations were developed for African American and White males and females, which included the following variables in the equations: age (years), concentration of TC (mg/dl) and HDL-C (mg/dl), treated or untreated systolic blood pressure (mmHg), diabetes status (yes/no), and self-reported current smoking status (yes/no). First, the natural log of age, TC, HDL-C, and systolic blood pressure were calculated with systolic blood pressure being either a treated or untreated value. Next, we multiplied these values by the coefficients from the estimated equation parameters of the Pooled Cohort Equations for the specific race-sex group of the population. The sum of the products of the previous calculations were then calculated for the population. Finally, we estimated the 10-year risk of ASCVD event as

$$Predicted ASCVD Risk= 1-S_{0}{(t)}^{e^{Ind ividual score-Mean score}}$$

The ASCVD risk was then calculated as 1 minus the survival rate at 10 years raised to the power of the exponent of the coefficient*value sum minus the race and sex specific overall mean coefficient*value sum (8). From these results, each member of the primary prevention population was assigned a probability of developing an ASCVD event, which was used to determine which participant was statin-eligible according to one of the three 10-year ASCVD risk thresholds we evaluated.

### *C.* Projection of ASCVD and T2D incidence and non-ASCVD mortality

Next, we calculated annual age- and sex-specific ASCVD, T2D, and non-ASCVD mortality transition probabilities by statin treatment. The transition probabilities were then used to project the expected number of incident ASCVD events, incident T2D, and number of non-ASCVD deaths annually in Markov models. As an example, calculation of the one-year transition probability for ASCVD stratified by statin use, sex and five-year age groups, was estimated as:

$${Rate of ASCVD}_{Non statin users}=\frac{{Rate ASCVD}_{overall}}{(\left( 1-P_{statin users} \right)+P_{statin users}*{RR}_{statin users}))}$$

and:

$${Rate ASCVD}_{statin users}={Rate ASCVD}_{Non statin users}* {RR}_{statin users}$$

where ${ASCVD}_{overall}$ was the one-year incidence rate of ASCVD, $P_{statin users}$ was the prevalence of statin users (to accommodate changes to statin adherence, which were updated each cycle [Supplemental Figure 1]), and ${RR}_{statin users}$ was the RR of ASCVD among statin users compared to non-statin users.

We then converted the ASCVD rate to the ASCVD transition probability as:

$${Probability of ASCVD}_{statin users}=1-e^{-{Rate ASCVD}_{statin users}*Time}$$

where *Time* corresponded to the number of years each cycle represented (1 cycle = 1 year).

### D. TreeAge Pro Software

The series of Markov models used in these analyses were implemented through the TreeAge Pro software (9). We used the healthcare model supported by TreeAge Pro to create decision trees for each intervention. Each decision node corresponded to a 5-year age group stratified by sex that included branches for each health state (T2D, ASCVD, mortality) (9). Once the Markov model was built, a cohort analysis was conducted to generate output by one-year cycles to identify incident cases (i.e. T2D and ASCVD). In addition, the TreeAge Pro software allowed us to check missing probabilities, missing states, and unused variables found in our Markov models to validate our models.

**LITERATURE CITED**

1. Centers for Disease Control and Prevention. National Health and Nutrition Examination Survey 2012 [Available from: <http://www.cdc.gov/nchs/nhanes.htm>.

2. Johnson CL, Paulose-Ram R, Ogden CL, Carroll MD, Kruszan-Moran D, Dohrmann SM, et al. National health and nutrition examination survey. Analytic guidelines, 1999-2010. 2013.

3. Howard VJ, Cushman M, Pulley L, Gomez CR, Go RC, Prineas RJ, et al. The reasons for geographic and racial differences in stroke study: objectives and design. Neuroepidemiology. 2005;25(3):135-43.

4. Friedewald WT, Levy RI, Fredrickson DS. Estimation of the concentration of low-density lipoprotein cholesterol in plasma, without use of the preparative ultracentrifuge. Clinical chemistry. 1972;18(6):499-502.

5. Longstreth WT, Jr. The REasons for Geographic And Racial Differences in Stroke (REGARDS) Study and the National Institute of Neurological Disorders and Stroke (NINDS). Stroke; a journal of cerebral circulation. 2006;37(5):1147.

6. Howard VJ, Kleindorfer DO, Judd SE, McClure LA, Safford MM, Rhodes JD, et al. Disparities in stroke incidence contributing to disparities in stroke mortality. Annals of neurology. 2011;69(4):619-27.

7. Goff DC, Jr., Lloyd-Jones DM, Bennett G, Coady S, D'Agostino RB, Gibbons R, et al. 2013 ACC/AHA guideline on the assessment of cardiovascular risk: a report of the American College of Cardiology/American Heart Association Task Force on Practice Guidelines. Circulation. 2014;129(25 Suppl 2):S49-73.

8. Muntner P, Colantonio LD, Cushman M, Goff DC, Howard G, Howard VJ, et al. Validation of the atherosclerotic cardiovascular disease Pooled Cohort risk equations. JAMA : the journal of the American Medical Association. 2014;311(14):1406-15.

9. TreeAge Pro.TreeAge Software. Inc Williamstown, MA. 2014.
