# Supplementary material for: Projections of incident atherosclerotic cardiovascular disease and incident type 2 diabetes across evolving statin treatment guidelines and recommendations: A modelling study
Source: PLoS Med. 2020 Aug 26;17(8):e1003280. doi: 10.1371/journal.pmed.1003280 (PMC7449387; doi:10.1371/journal.pmed.1003280)
Supplement: Attachment — Submitted filename: engedaResponseMay26_Final_Formatted.docx [file pmed.1003280.s015.docx]

May 26^th^, 2020

Emma Veitch, PhD
PLOS Medicine

On behalf of Clare Stone, PhD, Acting Chief Editor,
PLOS Medicine
[plosmedicine.org](http://plosmedicine.org)

**Re: PMEDICINE-D-19-04150**

Dear Drs. Stone and Veitch,

Thank you for providing us with the opportunity to respond to reviewer comments for our newly retitled manuscript “Projections of incident atherosclerotic cardiovascular disease and incident type 2 diabetes across evolving statin treatment guidelines and recommendations: A modelling study.” We thank the reviewers for their careful review of our work, which has undoubtedly strengthened this manuscript. As such, we have taken great care to respond to each comment and make accompanying changes in the text. The largest change to our manuscript is the addition of a probabilistic sensitivity analysis, as requested by reviewers 1 and 2. Our PSA also informed our response to reviewer 3. As shown in our manuscript and response to all three reviewers, the results of our PSA are consistent with our findings and one of our overall messages, i.e., that estimates in females were more uncertain than estimates in males or the total population. We believe this work remains important for readers of *PLOS Medicine,* particularly in light of research released last week suggesting a continued under-representation of females in clinical trials that support US Food and Drug Administration approval of new molecular entities (MS Khan et al., 2020).

Please do not hesitate to contact us if we may provide additional information. We look forward to your reply.

Sincerely,

Sincerely,

Dr. Christy Leigh Avery

Associate professor

Department of Epidemiology

Cardiovascular Disease Program

123 W. Franklin St., Suite 410

Chapel Hill, NC 27516

T: 919/270-0174

[Christy_avery@unc.edu](mailto:Christy_avery@unc.edu)

Requests from the editors:

*Please revise your title according to PLOS Medicine's style. Your title must be nondeclarative and not a question, beginning with the main concept. The study design (eg in this case - "modelling study," etc.) should appear in the subtitle (ie, after a colon).

*We have revised our title as requested: Projections of incident atherosclerotic cardiovascular disease and incident type 2 diabetes across evolving statin treatment guidelines and recommendations: A modelling study*

*Please structure your abstract using the PLOS Medicine headings (Background, Methods and Findings, Conclusions) -"methods and findings" should be a single subsection. Please also rephrase some of the sections so these are written as full sentences rather than sentence fragments ("to compare.." etc).

*The abstract has been restructured, as requested.*

*In the last sentence of the Abstract Methods and Findings section, please describe the main limitation(s) of the study's methodology.

*We have included the following sentence describing the study’s main limitation: “’* *This study’s primary limitation was uncertainty in estimates of statin associated T2D risk, highlighting areas where additional clinical and public health research is needed.”*

*At this stage, we ask that you include a short, non-technical Author Summary of your research to make findings accessible to a wide audience that includes both scientists and non-scientists. The Author Summary should immediately follow the Abstract in your revised manuscript. This text is subject to editorial change and should be distinct from the scientific abstract. Please see our author guidelines for more information: <https://journals.plos.org/plosmedicine/s/revising-your-manuscript#loc-author-summary>

*We now include a non-technical Author Summary.*

*Ideally please change the referencing format (this should be simple if referencing software has been used) to change citation callouts in the text to numbers in square brackets (ie, [1, 2] etc). Many thanks

*Our reference formatting has been changed.*

*Did your study have a prospective protocol or analysis plan? Please state this (either way) early in the Methods section.

a) If a prospective analysis plan (from your funding proposal, IRB or other ethics committee submission, study protocol, or other planning document written before analyzing the data) was used in designing the study, please include the relevant prospectively written document with your revised manuscript as a Supporting Information file to be published alongside your study, and cite it in the Methods section. A legend for this file should be included at the end of your manuscript.

b) If no such document exists, please make sure that the Methods section transparently describes when analyses were planned, and when/why any data-driven changes to analyses took place. 

c) In either case, changes in the analysis-- including those made in response to peer review comments-- should be identified as such in the Methods section of the paper, with rationale.

*Please see our changes to the text in on page 8, paragraph 1, where we describe the conceptualization and development of our simulation model.*

Comments from the reviewers:

Reviewer #1: The aim of this study by Engeda and colleagues was to investigate (and project) the potential impact of statin use in primary prevention, that is, reducing the risk of atherosclerotic cardiovascular disease (ASCVD) on the one hand, and the potential harm - risk of incident type 2 diabetes mellitus in a population of adult Americans aged 40 - 75 years. A Markov model was developed and used to compare the projected the impact (benefit and harms) on population groups stratified by 10-year ASCVD risk, based on American College of Cardiology (ACC)/ American Heart Association (AHA) guidelines.

Authors report that in the overall cohort, the number of averted ASCVD events outweigh the number incident type 2 diabetes mellitus cases from statin treatment for primary prevention, with a likelihood to help or harm of 2.26 - 2.90. The magnitude of this impact was greater in men and older people as opposed to women and younger people with more modest impact. Their findings are sensitive to the varied relative risks of statin use and risk of type 2 diabetes mellitus. Their findings are interesting as it provides insights to a less-well investigated relationship explored in the same population.

I have a few comments/questions on the methods and reporting of results that require clarification.

Methods

1) Page 6, "The first step in constructing the simulation model was to collect input data." I understand authors are referring to model development when they talk about 'constructing' a simulation model? If that be the case, it appears this does not quite sit well with the International Society for Pharmacoeconomics and Outcomes research (ISPOR) guidelines for good research practices (See: Med Decis Making. 2012 Sep-Oct;32(5):678-89.), which suggest that conceptualization and model development to answer the decision question should precede the search of input data. The model structure should therefore not [initially] be dictated by the range of available data but rather the other way around. Admittedly, this goes through an iterative process before finally adopting the final model. Authors might want to revise this statement to better reflect their process aligns with guidelines?

*To better reflect the iterative process guiding conceptualization and model development, we have updated the methods as follows (page 8, paragraphs 1 and 2): A simulation model was conceptualized and then developed to evaluate statin guidelines and recommendations because few available studies (1) were contemporary (2), spanned ages specified by current guidelines or recommendations, and (3) precisely and validly measured ASCVD and T2D incidence within generalizable male and female multi-ethnic populations with long-term follow-up [20]. This study did not consider costs associated with statins, T2D, or ASCVD… After conceptualizing the problem, the next step was to collect input data.*

2) Page 8, "Primary prevention statin-associated ASCVD RRs were obtained from the Cholesterol Treatment Trialists' meta-analysis of 22 trials (statin treatment versus control), from which we abstracted separate RR point estimates for males (RR=0.78) and females (RR=0.84).(28)" I have some questions in relation to this;

a) A good number of the trials included in this meta-analysis were conducted in the UK and Europe. Authors may want to comment on the transferability/applicability of these estimates to the U.S., given there's likely differences in the epidemiology of, and treatment of CVD events between these countries and the U.S.? Was there not pooled evidence from the literature with U.S specific data that could be used instead?

*We included two model inputs with non-U.S. data: meta-analyzed estimates of T2D-statin associations and meta-analyzed estimates of the statin-ASCVD association. For the former estimate, our previous meta-analysis did not detect significant heterogeneity by country of residence (P>0.05) ^1^. For the latter, we could not identify a meta-analysis that tested for heterogeneity in the statin-ASCVD effect by country of origin. We now describe this limitation in the discussion (page 23, bridging paragraph) “Fourth, two model inputs included non-U.S. data: meta-analyzed estimates of statin-T2D RRs and meta-analyzed estimates of the statin-ASCVD RRs. For the former estimate, our previous meta-analysis did not detect significant heterogeneity by country of residence (P>0.05), supporting pooling. For the latter estimate, we could not identify a meta-analysis that tested for heterogeneity by country of residence, although prior population-based studies conducted before widespread statin use suggested comparable LDL-C levels in European and North American adults. However, the degree to which heterogeneity from variation in the epidemiology of ASCVD by country of residence affects estimates of statin associated ASCVD RRs cannot be evaluated in depth ^2, 3^.”*

b) Authors purport to have extracted point estimates of relative risks for males and females from this study. Authors should clarify how this was done. In addition, it is unclear if there was any uncertainty e.g. 95% confidence intervals for these estimates. I would expect some uncertainty around the relative risk of events, which needs to be accounted for in the probabilistic sensitivity analysis, to get a comprehensive sense of the risk/impact. Could authors clarify how this was managed?

*We now state that published sex-specific point estimates were abstracted from the original meta-analysis ^4^ (page 10, paragraph 2), noting that we identified and corrected a typo that mis-specified the male RR as 0.78, not 0.64. Regarding uncertainty around the RRs, please see comment 7, which describes our new probabilistic sensitivity analysis that included uncertainty in the statin-ASCVD RR by sex.*

c) Relative risk of vascular events would vary by age, e.g. differ for 40-50yrs, 51-60yrs, 61-70yrs, etc. Using just a single point estimate of RR is likely to undermine this heterogeneity [at least by age]. In a later part of the results, authors discuss variation by age, but it is unclear or not immediately apparent how this was approached, at least, in terms of the relative risks. Could authors comment on this with respect to their modelling (possible limitation to be discussed)?

*We did not incorporate different statin-ASCVD RRs by age because results from the Cholesterol Treatment Trialists’ (CTT) Collaborators suggested that age at baseline did not significantly modify the effect of statins on major vascular events ^4^. Please see page 21, paragraph 1 where we highlight this finding.*

3) Page 9, Model overview: "For each annual cycle, statin-eligible populations could either remain alive and non-diseased or transition to having T2D, an ASCVD event, or a non-ASCVD death". Did the health state 'ASCVD event' include fatal and non-fatal cases? Otherwise, how were the fatal ASCVD events modelled?

*We updated Figure S1 and now clarify that ASCVD events include fatal and non-fatal cases (page 11, paragraph 1) “For each annual cycle, statin-eligible populations could either remain alive and non-diseased (i.e., without ASCVD or T2D) or transition to having T2D, an ASCVD event (fatal or non-fatal), or a non-ASCVD death (Figure S1).*

4) Regarding heterogeneity, it would be interesting to see if there was a differential in the impact of statin treatment on ASCVD events between Black Americans, Hispanic and non-Hispanic whites. In addition, variation in impact by socio-economic status (SES). I wonder if authors considered exploring these (at least in sensitivity analysis) as there are likely to be differences in risk by ethnicity and SES.

*We agree with the reviewer that it would be interesting to evaluate heterogeneity by race/ethnicity and SES. As now described on page 22, paragraph 1, the necessary input data are either unavailable (i.e., estimates of statin-associated changes in ASCVD and T2D incidence by race, ethnicity, and SES and ASCVD incidence among Hispanics subgroups) or would become too imprecise, as ASCVD and T2D incidence rates estimates are already stratified by sex and 5-year age group. We state “Second, we limited our study to projecting incidence in a primary prevention populations 40-75 years of age of non-Hispanic African Americans or White race/ethnicity given limited input data (e.g., T2D and ASCVD incidence) in other racial/ethnic group. Although this decision may limit generalizability, 73% of the U.S. population are non-Hispanic African American or White ^5^. We also reported projections in the total population and not by race/ethnicity, anticipating imprecise model inputs by race/ethnicity. Future work may wish to expand simulations to include additional populations and evaluate heterogeneity by race, ethnicity, or other potentially modifying factors.”*

5) The paper uses the REGARDS study for input data on T2D and ASCVD incidence. There are some issues with this:

a) Authors should clarify why the data from REGARDS was chosen in preference to other data. For example, there seems to be data available from the Centre for Disease Control (page 19 and reference 58).

*REGARDS data were chosen in lieu of national data from the Center for Disease Control (CDC) because the CDC measured diabetes incidence using an interviewer-administered questionnaire^6^. Previous research has shown that self-reported diabetes has moderate sensitivity^7^. Self-reported diabetes also cannot capture undiagnosed diabetes, which represents an estimated 10% of U.S. diabetes cases^8^. In contrast, the REGARDS study measured diabetes using plasma glucose measures and self-reported use of diabetic medications. We have updated the manuscript as follows (page 9, paragraph 1): “*In the absence of nationally representative data measuring ASCVD and T2D incidence using validated protocols capturing undiagnosed disease and mortality ^9^, we leveraged data from the ongoing Reasons for Geographic and Racial Differences in Stroke (REGARDS) study (data used: 2003-2015).”

b) While REGARDS is a national survey, it is unclear if it is nationally representative, given that the study aim was to include 30% of participants from the "Stroke Belt" states. Please confirm the study is nationally representative.

*We no longer describe the REGARDS study as a “national” study in an attempt to add clarity. We also now describe the rationale for selection of the REGARDS study, i.e., “In the absence of nationally representative data measuring ASCVD and T2D incidence using validated protocols capturing undiagnosed disease and mortality, we leveraged data from the REGARDS study” (page 9, paragraph 1).*

c) The REGARDS study only included participants from ages 45 years, but the paper starts with populations at age 40 and uses the data from the 45 - 50 age group for the 40 - 45 group and "assessed different specifications via meta analyses". Please clarify what this means.

*Because the REGARDS study only included participants aged ≥45 years and given the comparability of estimated ASCVD and T2D incidence rates between ages 45-60, we assigned ASCVD and T2D incidence rates estimated in the 45-50 year age group to the 40-44 year age group. To clarify this point, we have updated the footnote in S2 Table (page 7 of the supplement) and in the methods (pages 9-10, bridging paragraph) “Because the REGARDS study only included participants aged ≥45 years and given the comparability of estimated ASCVD and T2D incidence rates between ages 45-60, we assigned ASCVD and T2D incidence rates estimated in the 45-50 year age group to the 40-44 year age group.”*

6) The paper uses 10-year ASCVD risk to determine eligibility for statin treatment. This reflects the 2013 Guidelines but does not reflect the 2018 AHA/ACC Guideline on the Management of Blood Cholesterol (2018 Guidelines), which use other parameters to determine statin eligibility. Further, it is unclear whether the use of 10-year ASCVD risk to determine eligibility for statin treatment reflects current practice. Authors should provide information to demonstrate that the statin eligibility criteria used in the intervention groups reflect current practice. This is important to demonstrate that the population eligible for statins in the intervention groups accurately reflect those people that would be eligible for statins in the population.

*In response to this reviewer’s concern that “it is unclear whether the use of 10-year ASCVD risk to determine eligibility for statin treatment reflects current practice,” we note the following. Both the 2013 ^10^ and 2018 ^11^ guidelines suggest using the pooled cohort risk equation ^12^ to calculate ASCVD risk as a starting point for considering statin therapy among adults age 40-75 being evaluated for primary ASCVD prevention. The details of these guidelines are outlined below.*

*Both guidelines mention 5% and 7.5% as thresholds for treatment, while the US Preventive Services Task Force suggests a threshold of 10% for initiating statin therapy for primary prevention ^13^. Thus, we believe our use of 5, 7.5, and 10% thresholds for statin eligibility aligns appropriately with current guidelines on cut-points for initiating such therapy.*

*One of the authors (TCK) performed a literature search to assess use of thresholds to initiate therapy. One report indicates that 80% of clinicians always or very often use the 10 year risk score when considering the initiation of statin therapy ^14^. This author was unable to identify published data on the actual thresholds use by practicing clinicians. That said, TCK is an active primary care clinician in an academic practice and also precepts residents regularly. In considering statin therapy, almost all of his colleagues and residents use the 10-year risk score, which is automatically calculated by the electronic health record, as a starting point to initiate a discussion with patients about starting statin therapy for primary prevention.*

*Thus, we have modified the manuscript as follows (pages 22-24, bridging paragraph): “We also did not explicitly consider the ACC/AHA 2018 guideline given data limitations. However, both the 2013 ^10^ and 2018 ^11^ ACC/AHA guidelines use the Pooled Cohort Equation with 5% and 7.5% treatment thresholds as starting points for considering statin therapy among adults age 40-75 being evaluated for primary ASCVD prevention. The US Preventive Services Task Force suggests a threshold of 10% for initiating statin therapy for primary prevention ^13^. Thus, the 5%, 7.5%, and 10% thresholds for statin eligibility aligns appropriately with current guidelines and physician practice ^14^ when determining cut-points for initiating statin therapy.”*

7) The current analysis seems not to have performed an uncertainty analysis? I would assume authors would consider uncertainty distributions around key input parameters e.g. relative risk, to allow for a probabilistic sensitivity analysis?

*As requested by the reviewer, we now include a probabilistic sensitivity analysis. As described in the manuscript (page 13, bridging paragraph): “Finally, we examined the robustness of projections to variation in two sets of study input parameters (sex-specific statin-ASCVD RRs as well as sex- and age-specific ASCVD and T2D incidence rates) by performing three probabilistic sensitivity analyses (PSA). Our first two PSA considered uncertainty from each of the input parameters separately and the third PSA considered uncertainty from each input parameter simultaneously. For each PSA, we ran 1,000 replications that sampled from the probability distribution of each parameter, which were determined based on 95% confidence intervals (S2 Table). For ASCVD and T2D incidence rates, we assumed a normal distribution for the mean as the estimate. For the statin-ASCVD RR, we assumed a lognormal distribution for the RRs ^15^. Because the primary analyses already evaluated the influence of variation in statin-T2D RRs by considering three separate RRs, we did not consider this parameter in our PSA.” Our PSA echoes one of our major findings, i.e., (page 16, paragraph 1): Consistent with our findings, PSA indicated that estimates in females were more uncertain than estimates in males or the total population (****S7 Figure****).*

Results and discussion

8) Page 16, paragraph 1: The paper states "one ASCVD event would be prevented for every 155 - 197 adults treated" and "benefits of statin treatment were even more pronounced in males and older populations compared to females and younger populations". These statements rely on the NNB produced by the model. However, the results section does not disclose the NNB - it focuses on the changes in ASCVD events prevented between different guidelines and the changes to LHH between different statin-associated T2D RRs, sex and age groups. The authors should include in the results the NNB, including how this varies by age and sex, in order to support this part of the discussion.

*We added Figure S3 (page 12 in the supplement), which includes NNH and NNB estimates in the total population. Together with Figure S8 and accompanying edits to the text in the results section that , now provide information on how the NNB varies in the overall population and by age and sex.*

9) Page 20, paragraph 1: The paper states "In addition, we did not consider the 2018 guidelines given data limitations although we anticipate projections of these guidelines would fall between 5% and 7.5% ASCVD risk thresholds we considered, providing some degree of information." This sentence appears incomplete. Authors should clarify what information is provided. For example, do these projections provide information about the risk-benefit analysis under the 2018 Guidelines? The authors should clarify the basis of their anticipated projections - how were they reached? The cited reference (62) does not seem to support the statement and is relevant to the previous point (comparison of other published guidelines to the 2013 Guidelines).

*We agree with the reviewer and have removed our speculation about what the 2018 guidelines would show. Instead, we now more explicitly describe how both the 2013 and 2018 guidelines use Pooled Cohort Equation-derived treatment thresholds as starting points for considering statin therapy (page 22-24, bridging paragraph). We also did not explicitly consider the ACC/AHA 2018 guideline given data limitations. However, both the 2013 ^10^ and 2018 ^11^ ACC/AHA guidelines use the Pooled Cohort Equation with 5% and 7.5% treatment thresholds as starting points for considering statin therapy among adults age 40-75 being evaluated for primary ASCVD prevention.*

Minor comments and considerations

* The calculation of statin eligibility for the intervention groups relies upon calculation of the 10-year ASCVD risk using the Pooled Cohort Equation. The Equation is used to estimate 10-year ASCVD risk for an individual. Authors explain in the supplementary material (page 3) how they calculate a 10-year ASCVD risk at population level. The authors should clarify that section to make clear how this is achieved and avoid confusion.

*In an attempt to add clarity, we updated the supplement (page 4, paragraphs 4-6) describing that the predicted ASCVD risks were estimated for each member of the primary prevention population.*

* Page 6, end of first paragraph: The reference should be amended to include reference 3 and 4 because the sentence refers to both the 2013 and 2018 Guidelines.

*We have updated this sentence to include both references 3 and 4 (page 8, paragraph 2): “After conceptualizing the problem, the next step was to collect input data. In an attempt to maximize generalizability, we prioritized studies that included multi-ethnic (non-Hispanic African American and non-Hispanic White; capturing 73% of the U.S. population^5^) male and female statin-eligible adults aged 40-75 years, reflecting the ages specified by the AHA/ACC 2013 and 2018 guidelines ^10, 11^.”*

* Page 9, paragraph 2 & page 10, paragraph 2: Can authors provide some justification for their choice of adherence rates? including those in the sensitivity analyses. The cited study (reference 34) aimed to identify factors that predict adherence to statins rather than the adherence rates. However, it does not seem to include data about adherence rates (page 1413 of reference 34). Authors may want to provide clarification on how this reference informed their adherence rates.

*Reference 34 informed us of the wide variation observed in studies investigating statin adherence after initiation. Given the wide variation in assumed statin adherence found in the literature, we examined statin adherence under multiple assumptions. In the main analysis, we assumed an absolute annual decrease through year four and then assumed adherence would stabilize; however, we also examined statin adherence assuming three different annual relative decreases in statin use in our sensitivity analysis. Although (and as expected) the absolute values of ASCVD and T2D events changes in response to different assumptions underlying statin adherence, overall relative findings remained unchanged. In response to this reviewer’s comment, we have updated the methods section as follows (page 12, paragraph 2): “Given the wide variation in assumed statin adherence found in the literature, we examined statin adherence further in sensitivity analyses”.*

* Page 16, paragraph 1: "overall, these results suggest that further efforts are needed to more precisely characterize populations for whom statin treatment may introduce a large burden of adverse effects". Authors should clarify this sentence to provide a more specific conclusion that follows from the preceding sentences. The problem alluded to in the preceding sentence is the lack of a precise estimate of statin associated T2D risk. If that is correct, a more pertinent conclusion might be that further efforts are required to estimate this risk.

*We have updated this sentence as follows (page 19, paragraph 1): “Overall, these results suggest that further efforts are needed to better quantify statin-associated T2D risk across a range of populations, particularly female and younger populations for whom statin treatment may introduce a large relative burden of adverse events.”*

* Page 16, paragraph 2: "particularly of interest are scenarios where the number of incidence cases of T2D incurred were greater than, sometimes by several orders of magnitude, the number of ASCVD events prevented". The author should clarify whether they are referring to scenarios produced by this study and, if so, which scenarios.

*We have updated this sentence as follows (page 19, paragraph 2): “Particularly of interest are scenarios assuming higher statin associated T2D RR that compared females versus males and younger versus older populations. In these scenarios, the number of incident cases of T2D incurred were greater than, sometimes by several orders of magnitude, the number of ASCVD events prevented.”*

* Page 19 - 20: In response to the limitation of not using multiple guidelines, authors state: "recent reports have suggested overlap between guidelines considered herein with other published guidelines. For example, comparable estimated NNTs have been reported" between other published guidelines and those used by the study. To demonstrate the suggested overlap, the authors may want to consider comparing these other published guidelines and the 2013 Guidelines by reference to the populations that would be eligible for statin therapy rather than the effects of statin therapy (i.e. NNTs). For example, to compare the 2018 Guidelines and the 2013 Guidelines, the authors refer to projections of the ASCVD risk thresholds produced by the 2018 Guidelines. Perhaps a similar projection could be made for the other published guidelines.

*As requested, we now state (page 22-24, bridging paragraph) that both the 2013 ^10^ and 2018 ^11^ ACC/AHA guidelines use the Pooled Cohort Equation with 5% and 7.5% treatment thresholds as starting points for considering statin therapy among adults age 40-75 being evaluated for primary ASCVD prevention. Thus, the eligible populations for these two guidelines are the same.*

Supplementary material

1) Authors state, "The definition of stroke in the REGARDS study was: Prevalent stroke was defined as a positive response to either "Were you ever told by a physician that you had a stroke?" or "Were you ever told by a physician you had a mini-stroke or TIA, also known as a transient ischemic attack?" I anticipate that the risk of TIA and the risk of ischaemic stroke are different. Any considerations in this risk differential?

*In order to add clarity and emphasize that the above-quoted text refers to exclusion criteria, we have updated the supplemental material as follows (page 4, paragraph 1): Prevalent stroke, which was used as an exclusion criterion when estimating ASCVD incidence, was defined as a positive response to either “Were you ever told by a physician that you had a stroke?” or “Were you ever told by a physician you had a mini-stroke or TIA, also known as a transient ischemic attack?”^16^. Please note that stroke incidence was determined in accordance with the World Health Organization definition.*

2) In S4 Table, frequencies seem to be in parenthesis and percentage out, contrary to what authors refer to as for example, Male (%). Please, consider adjusting.

*We have adjusted the heading of S4 Table (page 9 of supplement) to show frequencies in parenthesis and percentages out as follows: Male % (N).*

References

Reference 15, 28 and 58 and 62 seem incomplete.

*We have updated the references as follows:*

*Engeda JC, Stackhouse A, White M, Rosamond WD, Lhachimi SK, Lund JL, et al. Evidence of heterogeneity in statin-associated type 2 diabetes mellitus risk: A meta-analysis of randomized controlled trials and observational studies. Diabetes research and clinical practice. 2019;151:96-105. Epub 2019/04/08. doi: 10.1016/j.diabres.2019.04.005. PubMed PMID: 30954511; PubMed Central PMCID: PMCPMC6544490.*

*Mihaylova B, Emberson J, Blackwell L, Keech A, Simes J, Barnes EH, et al. The effects of lowering LDL cholesterol with statin therapy in people at low risk of vascular disease: meta-analysis of individual data from 27 randomised trials. Lancet. 2012;380(9841):581-90. Epub 2012/05/23. doi: 10.1016/s0140-6736(12)60367-5. PubMed PMID: 22607822; PubMed Central PMCID: PMCPMC3437972.*

*Centers for Disease Control and Prevention. National diabetes statistics report, 2020. Atlanta, GA: Centers for Disease Control and Prevention, US Department of Health and Human Services. 2020.*

*Mortensen MB, Nordestgaard BG. Statin Use in Primary Prevention of Atherosclerotic Cardiovascular Disease According to 5 Major Guidelines for Sensitivity, Specificity, and Number Needed to Treat. JAMA Cardiol. 2019. Epub 2019/10/03. doi: 10.1001/jamacardio.2019.3665. PubMed PMID: 31577339; PubMed Central PMCID: PMCPMC6777225.*

-----------------------------------------------------------

Reviewer #2: This is a well conducted study on the projections of incident ASCVD and incident type 2 diabetes using different assumptions of statin treatment recommendations. The study design, datasets, statistical methods and analyses, and presentation (tables and figures) and interpretation of the results are mostly adequate. However, there are still a few issues needing attention.

1) This is a simulation study using the Markov model, popular in the health economics field, which is fine. It's based on many assumptions and also the parameters estimated from the existing datasets. The key question is the believability and robustness of the model, which we don't really know. Are there any validation procedures for the proposed models? In other words, to what degree, should we trust the model that can reflect what's going on in the real world and in the future?

*We agree with the reviewer that the validity of this study depends on the believability and robustness of the model. As emphasized in our paper, we limited our study to a brief time span (10 years) and described our results as projections, not predictions. Our results also were consistent with findings of Yebyo et al., (2019) ^17^, who reported strong heterogeneity by sex and age for statin-associated net benefit. However, we emphasize that findings from Yebyo et al., (2019) are not directly comparable with those presented herein because they considered harms in addition to T2D, were largely based on data from a Swiss population, used a different modeling approach, and reported different parameters, among other differences. In an attempt to more systematically evaluate model robustness, we now include a probabilistic sensitivity analysis, described as follows (page 13, bridging paragraph): “Finally, we examined the robustness of projections to variation in two sets of study input parameters (sex-specific statin-ASCVD RRs as well as sex- and age-specific ASCVD and T2D incidence rates) by performing three probabilistic sensitivity analyses (PSA). Our first two PSA considered uncertainty from each of the input parameters separately and the third PSA considered uncertainty from each input parameter simultaneously. For each PSA, we ran 1,000 replications that sampled from the probability distribution of each parameter, which were determined based on 95% confidence intervals (S2 Table). For ASCVD and T2D incidence rates, we assumed a normal distribution for the mean as the estimate. For the statin-ASCVD RR, we assumed a lognormal distribution for the RRs ^15^. Because the primary analyses already evaluated the influence of variation in statin-T2D RRs by considering three separate RRs, we did not consider this parameter in our PSA.” Our PSA echoes one of our major findings, i.e., (page 16, paragraph 1): Consistent with our findings, PSA indicated that estimates in females were more uncertain than estimates in males or the total population (****S7 Figure****).*

2) Although the Markov model can be used for simulation, it seems a bit simplistic. A few issues remains. Firstly, it's highly likely the model will rely on the projected prevalence of ASCVD and T2D in the next 10 years. Not sure how exactly the authors did it. It seems that they used the REGARDS study data to estimate? Then what prediction models were used for the projection, linear, Poisson or exponential? They are many ways to predict the prevalence using past and future information based on different models. Some good examples can be seen from the latest Global Burden of Disease (GBD) studies. Robust and reliable estimates of future prevalence of the events will have a big impact on the simulation results, and at least this should be discussed in the limitation.

*We apologize for the confusion. In our simulation model, we begin with the currently statin eligible population (see Table 1). For this population, we project the number of incident cases of ASCVD and T2D in one-year increments. Said another way, we project the number of cases given our current knowledge about input parameters ^18^. We explicitly do not “predict” the future incidence of T2D or ASCVD in the total population, as predictions would require a much more complex and uncertain simulation model accounting for factors such as migration, trends in mortality, progress in medical technology, or economic growth. In an attempt to add clarity, we have modified our conceptual model (S1 Figure) and added the following sentence to the methods (pages 11-12, bridging paragraph): “As our results project only the incident events for the primary prevention population over ten years, we cannot make a statement about the population level ASCVD or T2D incidence in ten-years’ time, as this estimate may depend on other factors outside of the scope of our model, e.g. migration patterns or progress in medical technology.”*

Secondly, in the Markov model, for different transition states, what happens if a person is still alive but developed diseases other than ASCVD or T2D later? It seems they were not assigned to any state in the study.

*We have updated Figure S1 as well as the methods (page 10, paragraph 1): “We did not simulate other disease states other than non-ASCVD death under our assumption that only the incidence of ASCVD and T2D were associated with statin use.”*

3) The presentation of Table 1 needs to improve and to use standard format. For continuous variables such as age and total cholesterol, they should be summarised as mean and SD if normal distribution, or median and IQR if non-normal distribution. For categorical data, they need to be summarised as count and percentage.

*Table 1 has been updated, as requested (page 14).*

-----------------------------------------------------------

Reviewer #3:

Projections of incident atherosclerotic cardiovascular disease and incident type 2 diabetes across evolving statin treatment guidelines and recommendations

In this study, Dr Engeda and co-workers have estimated the CVD risk reduction from statins used against induced Type 2 diabetes in adults free of prior CVD, eligible to statins use according to contemporary guidelines. Using simulation models (Markov models) they found that the number of incident CVD averted was at least twice as higher the number of incident diabetes induced. Women and younger adults had the lowest absolute benefits and the investigators concluded that these were areas in need for additional clinical and public health research.

While the question investigated has merit, the approach used (simulation), which is essentially based on assumptions, has limitations, many of which are highlighted by the investigators. Where the study really falls short in my view is that the investigators should have included possible scenario to mitigate diabetes risk from statin use. Just like they have used absolute risk model to predict CVD risk, similar models exist to predict incident diabetes risk. Furthermore, lifestyles and other interventions the reduced the unset of diabetes in people at risk are also known. The investigators could therefore consider accounting for the impact interventions to prevent diabetes in those at high risk at baseline, on the future risk of type 2 diabetes in people started on statins. The reality is that, in spite of the authors' findings, chances of withholding statins in eligible patients because of the fear of diabetes risk are very low, and therefore additional focus should be on how to mitigate the risk of diabetes in people started on statin.

*We thank the reviewer for this important observation. The results of our simulation show the effect of three plausible effect estimates of statin use on T2D incidence at the population-level. We believe that investigating interventions that lower the risk of T2D incidence among statin-users is the next stage in research (and by extension, simulation). However, at the current stage it is not straightforward to incorporate such intervention scenarios in a simulation. As an example, such a simulation would require robust data on T2D risk-lowering interventions not only in the overall population, but also by statin use and within the subgroups we examined. However, it is currently unclear if a generally accepted T2D risk lowering intervention (say, increase physical exercise by 50 minutes per week) has the same effect in statin users as in non-statin-users. It might very well be that there is an interaction between, say, physical exercise and statin use and, hence, the risk in statin users may or may not be lowered by the same magnitude as in non-statin-users. In principle, it might be possible that certain proven interventions may have no or a much lower effect (or perhaps even a higher effect) among statin-users than non-statin-users in preventing T2D. There also could be differences by age and sex. Hence, incorporating such scenarios at the current stage would be speculative and it would make our (deliberately parsimonious) simulation less robust.*

Other considerations: Incident diabetes was based on history, fasting or random glucose, which is not optimal.

*We agree that basing incident diabetes on history and fasting or random glucose is of reduced accuracy when compared to oral glucose tolerance tests ^19^. Importantly, results of our newly performed PSA suggest that our results are robust to in uncertainty in estimates of T2D incidence (S7 Figure).*

**REFERENCES**

1.	Engeda JC, Stackhouse A, White M, Rosamond WD, Lhachimi SK, Lund JL, Keyserling TC and Avery CL. Evidence of heterogeneity in statin-associated type 2 diabetes mellitus risk: A meta-analysis of randomized controlled trials and observational studies. *Diabetes research and clinical practice*. 2019;151:96-105.

2.	Carroll MD, Lacher DA, Sorlie PD, Cleeman JI, Gordon DJ, Wolz M, Grundy SM and Johnson CL. Trends in serum lipids and lipoproteins of adults, 1960-2002. *JAMA : the journal of the American Medical Association*. 2005;294:1773-1781.

3.	Finch S, Doyle W, Lowe C, Bates C, Prentice A, Smithers G and Clarke P. *National Diet and Nutrition Survey: people aged 65 years and over*: Stationery Office; 1998.

4.	Mihaylova B, Emberson J, Blackwell L, Keech A, Simes J, Barnes EH, Voysey M, Gray A, Collins R and Baigent C. The effects of lowering LDL cholesterol with statin therapy in people at low risk of vascular disease: meta-analysis of individual data from 27 randomised trials. *Lancet*. 2012;380:581-90.

5.	US Census Bureau Population Estimates. Vintage 2012: Downloadable Data Files.

6.	Centers for Disease Control and Prevention. National diabetes statistics report, 2020. *Atlanta, GA: Centers for Disease Control and Prevention, US Department of Health and Human Services*. 2020.

7.	Schneider AL, Pankow JS, Heiss G and Selvin E. Validity and reliability of self-reported diabetes in the atherosclerosis risk in communities study. *American journal of epidemiology*. 2012;176:738-743.

8.	Selvin E, Wang D, Lee AK, Bergenstal RM and Coresh J. Identifying trends in undiagnosed diabetes in US adults by using a confirmatory definition: a cross-sectional study. *Annals of internal medicine*. 2017;167:769-776.

9.	Virani SS, Alonso A, Benjamin EJ, Bittencourt MS, Callaway CW, Carson AP, Chamberlain AM, Chang AR, Cheng S and Delling FN. Heart disease and stroke statistics-2020 update: a report from the American Heart Association. *Circulation*. 2020;141:e139-e596.

10.	Stone NJ, Robinson JG, Lichtenstein AH, Bairey Merz CN, Blum CB, Eckel RH, Goldberg AC, Gordon D, Levy D, Lloyd-Jones DM, McBride P, Schwartz JS, Shero ST, Smith SC, Jr., Watson K, Wilson PW and American College of Cardiology/American Heart Association Task Force on Practice G. 2013 ACC/AHA guideline on the treatment of blood cholesterol to reduce atherosclerotic cardiovascular risk in adults: a report of the American College of Cardiology/American Heart Association Task Force on Practice Guidelines. *Journal of the American College of Cardiology*. 2014;63:2889-934.

11.	Grundy SM, Stone NJ, Bailey AL, Beam C, Birtcher KK, Blumenthal RS, Braun LT, de Ferranti S, Faiella-Tommasino J and Forman DE. 2018 AHA/ACC/AACVPR/AAPA/ABC/ACPM/ADA/AGS/APhA/ASPC/NLA/PCNA guideline on the management of blood cholesterol: a report of the American College of Cardiology/American Heart Association Task Force on Clinical Practice Guidelines. *Journal of the American College of Cardiology*. 2019;73:e285-e350.

12.	Goff DC, Jr., Lloyd-Jones DM, Bennett G, Coady S, D'Agostino RB, Sr., Gibbons R, Greenland P, Lackland DT, Levy D, O'Donnell CJ, Robinson JG, Schwartz JS, Shero ST, Smith SC, Jr., Sorlie P, Stone NJ, Wilson PW and American College of Cardiology/American Heart Association Task Force on Practice G. 2013 ACC/AHA guideline on the assessment of cardiovascular risk: a report of the American College of Cardiology/American Heart Association Task Force on Practice Guidelines. *Journal of the American College of Cardiology*. 2014;63:2935-59.

13.	Bibbins-Domingo K, Grossman DC, Curry SJ, Davidson KW, Epling JW, García FA, Gillman MW, Kemper AR, Krist AH and Kurth AE. Statin use for the primary prevention of cardiovascular disease in adults: US Preventive Services Task Force recommendation statement. *JAMA : the journal of the American Medical Association*. 2016;316:1997-2007.

14.	Clough JD, Martin SS, Navar AM, Lin L, Hardy NC, Rogers U and Curtis LH. Association of Primary Care Providers' Beliefs of Statins for Primary Prevention and Statin Prescription. *J Am Heart Assoc*. 2019;8:e010241.

15.	Briggs AH, Claxton K and Sculpher MJ. *Decision modelling for health economic evaluation*: Oxford University Press, USA; 2006.

16.	Howard VJ, Cushman M, Pulley L, Gomez CR, Go RC, Prineas RJ, Graham A, Moy CS and Howard G. The reasons for geographic and racial differences in stroke study: objectives and design. *Neuroepidemiology*. 2005;25:135-143.

17.	Yebyo HG, Aschmann HE and Puhan MA. Finding the Balance Between Benefits and Harms When Using Statins for Primary Prevention of Cardiovascular Disease: A Modeling Study. *Annals of internal medicine*. 2019;170:1-10.

18.	Bray D and von Storch H. “Prediction” or “Projection”?:The Nomenclature of Climate Science. *Science Communication*. 2009;30:534-543.

19.	Patel P and Macerollo A. Diabetes mellitus: diagnosis and screening. *American family physician*. 2010;81:863-70.
