# Supplementary material for: Projections of incident atherosclerotic cardiovascular disease and incident type 2 diabetes across evolving statin treatment guidelines and recommendations: A modelling study
Source: PLoS Med. 2020 Aug 26;17(8):e1003280. doi: 10.1371/journal.pmed.1003280 (PMC7449387; doi:10.1371/journal.pmed.1003280)
Supplement: Attachment — Submitted filename: engedaResponseJuly16_Final_Formatted.docx [file pmed.1003280.s016.docx]

Requests from the editors:

*Abstract-please add summary demographics for the cohort.

*Demographics are included in the description for the primary prevention population as follows (page 3): A total of 61,125,042 U.S. adults (58.5% female; 89.4% White; mean age=54.7 years) composed our primary prevention population, among whom 13-28 million adults were eligible for statin initiation.*

* Please tone down “We also found that females and the youngest adults received lower absolute benefits of statin treatment when compared to males and the oldest adults” to reflect that this is a finding from a modelling study. I suggest “found” is replaced with “our models suggest”.

*The author summary has been updated, as requested.*

* The Data statement requires revision, as you say all data are available but then you say “Some data are available from https://www.cdc.gov/nchs/nhanes/index.htm The remaining data cannot be shared publicly because they are from the REGARDS…”. Can you please clarify within the data statement which data can and cannot be accessed freely or otherwise. Please note that authors cannot be the contact persons for data requests and must be deposited with a data committee or an ethics committee or similar. For each data source used in your study:

a) If the data are freely or publicly available, note this and state the location of the data: within the paper, in Supporting Information files, or in a public repository (include the DOI or accession number).

b) If the data are owned by a third party but freely available upon request, please note this and state the owner of the data set and contact information for data requests (web or email address). Note that a study author cannot be the contact person for the data.

c) If the data are not freely available, please describe briefly the ethical, legal, or contractual restriction that prevents you from sharing it. Please also include an appropriate contact (web or email address) for inquiries (again, this cannot be a study author).

*We have updated the data statement, as requested. In addition, we have updated the text in our supplement as follows (page1, paragraph 3): “NHANES is a series of cross-sectional surveys and physical examinations conducted biennially to assess the health and nutritional status of the U.S. population and is publically available at* [*https://wwwn.cdc.gov/nchs/nhanes/Default.aspx*](https://wwwn.cdc.gov/nchs/nhanes/Default.aspx) (1).” *To describe REGARDS, we added (page 1, paragraph 5): “To gain access to REGARDS data, a manuscript proposal was submitted to* [*REGARDSAdmin@uab.edu*](mailto:REGARDSAdmin@uab.edu)*.”*

*The link to REGARDS researchers and institutions in the acknowledgements is broken. Please correct as needed.

*The link has been updated as follows (page 25, Acknowledgements section): “A complete list of participating REGARDS investigators and institutions can be found at* [*https://www.uab.edu/soph/regardsstudy/*](https://www.uab.edu/soph/regardsstudy/)*.”*

*The four guidelines need to be clearly highlighted in the methods as it currently doesn’t stand out

*The four guidelines are now in bold to highlight them in the methods.*

*The data sources are mentioned in suppl methods but brief details in main methods needed

*Details are added to the description of our data sources as follows (page 9,paragraph 3):	“NHANES collects demographic, nutritional, and health status information on a	nationally representative probability sample of the U.S. civilian population instituted by	the National Center for Health Statistics.”*

*Please can you provide p-values throughout, as needed. Note that we require exact p values, unless p<0.001

*Our simulation models do not generate p-values, however, we ran probabilistic sensitivity analyses to determine credible intervals and examine the robustness of our projections, as suggested previously.*

*Could you replace the word “harm” with an alternative, such as adverse effects or side effects ? The same goes for benefits, perhaps replace with effects?

*In order to add clarity, we replaced the word “benefit” with “treat” in the number needed to benefit estimate, to now read as number needed to treat throughout the manuscript. Because the word “harm” responds directly to the estimates we calculate (i.e. number needed to harm and likelihood to be helped or harmed) we believe harm is important for the manuscript(2, 3).*

*References should be in Vancouver style please

*References have been updated, as requested.*

*The STROBE checklist must be called out in the methods and please remove page numbers from the checklist as these are subject to change. Instead please use paragraphs and sections.

*The Methods section has been updated as follows (page 9, paragraph 1): “This study has been performed according to the STROBE checklist”. In addition, the STROBE checklist has been updated with page numbers removed and replaced with paragraphs and sections.*

* Did your study have a prospective protocol or analysis plan? Please state this (either way) early in the Methods section.

a) If a prospective analysis plan (from your funding proposal, IRB or other ethics committee submission, study protocol, or other planning document written before analyzing the data) was used in designing the study, please include the relevant prospectively written document with your revised manuscript as a Supporting Information file to be published alongside your study, and cite it in the Methods section. A legend for this file should be included at the end of your manuscript.

b) If no such document exists, please make sure that the Methods section transparently describes when analyses were planned, and when/why any data-driven changes to analyses took place.

c) In either case, changes in the analysis-- including those made in response to peer review comments-- should be identified as such in the Methods section of the paper, with rationale.

*The Methods section has been updated to specify when the simulation model was conceptualized as follows (page 9, paragraph 1): “A simulation model was conceptualized and then developed in September 2018 to evaluate statin guidelines and recommendations because few available studies (1) were contemporary, (2) spanned ages specified by current guidelines or recommendations, and (3) precisely and validly measured ASCVD and T2D incidence within generalizable male and female multi-ethnic populations with long-term follow-up (4). This study did not consider costs associated with statins, T2D, or ASCVD.”*

Comments from Reviewers:

* In your Author summary, Page 4, you write: "Projected differences by age and sex also were become more pronounced as the effect of statins on T2D was increased". You may want to delete either "were" or "become".

*The Author summary has been updated, as requested. The Author summary now states “Projected differences by age and sex also were become more pronounced as the effect of statins on T2D was increased.”*

**REFERENCES**

1.	Centers for Disease Control and Prevention. National Health and Nutrition Examination Survey 2012 [Available from: <http://www.cdc.gov/nchs/nhanes.htm>.

2.	Sedgwick P. Measuring the detriment of treatment: number needed to harm. Bmj. 2015;350:h2763.

3.	Laupacis A, Sackett DL, Roberts RS. An assessment of clinically useful measures of the consequences of treatment. The New England journal of medicine. 1988;318(26):1728-33.

4.	Walker AM. Confounding by indication. Epidemiology. 1996;7(4):335-6.
